# Genome and Ecology of a Novel *Alteromonas* Podovirus, ZP6, Representing a New Viral Genus, *Mareflavirus*

**DOI:** 10.1128/Spectrum.00463-21

**Published:** 2021-10-13

**Authors:** Ziyue Wang, Fang Zhang, Yantao Liang, Kaiyang Zheng, Chengxiang Gu, Wenjing Zhang, Yundan Liu, Xinran Zhang, Hongbing Shao, Yong Jiang, Cui Guo, Hui He, Hualong Wang, Yeong Yik Sung, Wen Jye Mok, Li Lian Wong, Jianfeng He, Andrew McMinn, Min Wang

**Affiliations:** a College of Marine Life Sciences, Frontiers Science Center for Deep Ocean Multispheres and Earth System, Institute of Evolution and Marine Biodiversity, Ocean University of China, Qingdao, China; b The Key Laboratory for Polar Science of State Ocean Administration, Polar Research Institute of China, Shanghai, China; c UMT-OUC Joint Center for Marine Studies, Qingdao, China; d Institute of Marine Biotechnology, Universiti Malaysia Terengganu (UMT), Kuala Nerus, Malaysia; e Institute for Marine and Antarctic Studies, University of Tasmania, Hobart, Tasmania, Australia; f The Affiliated Hospital of Qingdao University, Qingdao, China; University of Pittsburgh School of Medicine

**Keywords:** bacteriophage, *Alteromonas*, genomic and comparative genomic analysis, phylogenetic analysis, distribution

## Abstract

*Alteromonas* is a ubiquitous, abundant, copiotrophic and phytoplankton-associated marine member of the *Gammaproteobacteria* with a range extending from tropical waters to polar regions and including hadal zones. Here, we describe a novel *Alteromonas* phage, ZP6, that was isolated from surface coastal waters of Qingdao, China. ZP6 contains a linear, double-stranded, 38,080-bp DNA molecule with 50.1% G+C content and 47 putative open reading frames (ORFs). Three auxiliary metabolic genes were identified, encoding metal-dependent phosphohydrolase, diaminopurine synthetase, and nucleotide pyrophosphohydrolase. The first two ORFs facilitate the replacement of adenine (A) by diaminopurine (Z) in phage genomes and help phages to evade attack from host restriction enzymes. The nucleotide pyrophosphohydrolase enables the host cells to stop programmed cell death and improves the survival rate of the host in a nutrient-depleted environment. Phylogenetic analysis based on the amino acid sequences of whole genomes and comparative genomic analysis revealed that ZP6 is most closely related to *Enhodamvirus* but with low similarity (shared genes, <30%, and average nucleotide sequence identity, <65%); it is distinct from other bacteriophages. Together, these results suggest that ZP6 could represent a novel viral genus, here named *Mareflavirus*. Combining its ability to infect *Alteromonas*, its harboring of a diaminopurine genome-biosynthetic system, and its representativeness of an understudied viral group, ZP6 could be an important and novel model system for marine virus research.

**IMPORTANCE** *Alteromonas* is an important symbiotic bacterium of phytoplankton, but research on its bacteriophages is still at an elementary level. Our isolation and genome characterization of a novel *Alteromonas* podovirus, ZP6, identified a new viral genus of podovirus, namely, *Mareflavirus*. The ZP6 genome, with a diaminopurine genome-biosynthetic system, is different from those of other isolated *Alteromonas* phages and will bring new impetus to the development of virus classification and provide important insights into novel viral sequences from metagenomic data sets.

## INTRODUCTION

Viruses play a vital role in the control of marine microbial communities (1, 2) and are responsible for most prokaryote deaths. Recent studies using electron microscopy, epifluorescence microscopy, and flow cytometry have shown that viruses are the most abundant biological entities in diverse marine environments (3–5). They are not only highly abundant but also have very high genetic diversity (6). They replicate through infection of their hosts, which include both heterotrophic and autotrophic organisms. Some viruses can even change the genomes of marine organisms, regulate nutrient cycles, and facilitate evolution (1, 7). As the largest source of genetic elements on earth, viruses are thought to be responsible for most gene transfer in the oceans (8).

During the past decade, metagenomic studies of viruses have extended our understanding of marine viral community structure and function (9, 10). A total of 195,728 viral populations were assembled and described in the Global Ocean Viromes 2.0 (GOV 2.0) data set (9). However, most viral populations in these viromes cannot be either classified into known viral groups or associated with their hosts. Because phages (i.e., bacterial viruses) are believed to be the most abundant marine viruses, the isolation and genomic analysis of individual viruses, especially phages infecting dominant bacterial clades, such as pelagiphages, cyanophages, and *Puniceispirillum* phage HMO-2011, infecting the SAR116 bacterial clade, has substantially improved our understanding of the ecological and evolutionary roles of marine viruses and the interpretation of the virome sequences (11, 12). However, very few viruses from the dominant marine bacterial clades have been isolated.

*Alteromonas* species, which are widespread marine copiotrophs of the class *Gammaproteobacteria*, are commonly found in waters from the tropics to the poles, including hadal zones (13, 14). The *Tara* Oceans expedition found that *Alteromonas* had an occurrence rate reaching 80% with consistently high relative abundances (15). In the Challenger Deep of the Mariana Trench, the deepest site in the Earth’s oceans, *Alteromonas* was found to still be abundant in the hadal zone, at depths greater than 10,000 m below the surface (10 to 20% of 16S rRNA genes) (16). In phytoplankton blooms, the contribution of *Alteromonas* to the total bacterial biomass is at levels similar to those of the *Cytophaga-Flavobacteria-Bacteroides* group and *Roseobacter* (17–19).

*Alteromonas* has been found to make significant contributions to iron metabolism (20, 21) and play an important role in marine organic carbon and nitrogen cycling (22). In laboratory cocultures with cyanobacteria like *Prochlorococcus*, *Synechococcus*, and *Trichodesmium* (23), *Alteromonas* has demonstrated broad substrate preferences and can utilize dissolved organic carbon and particulate organic carbon supplied by photoautotrophs (24). Some isolates of *Alteromonas* have been used to synthesize exopolysaccharides (EPS) for production of colloidal suspensions of silver nanoparticles (AgNPs) (25, 26), which have excellent application prospects in nanomedicine, pharmaceutical science, and biomedical engineering (27, 28).

However, although *Alteromonas* plays important roles in the ocean, our understanding of *Alteromonas* phages is still poor. So far, only 11 *Alteromonas* phages have been isolated and deposited into GenBank, including five siphoviruses, four podoviruses, two autographiviruses, one myovirus, and one unclassified virus. The five *Siphoviridae* phages, including JH01, P24, PB15, XX1924, and vB_AcoS-R7M (29–32), were all isolated from the coastal water of China. Among those siphoviruses, vB_AcoS-R7M was found to share a set of similar characteristics with a number of siphophages infecting diverse aquatic opportunistic copiotrophs and inspired the creation of a new subfamily, *Queuovirinae* (32). *Podoviridae* phages vB_AmaP_AD45 P1 to P4 (33) have very similar genomic structures. They are similar to the N4-like *Podoviridae* genus but lack three common genes of the N4-like phages, encoding a cysteine-free N4-like virion-encapsidated RNA polymerase, a protein similar to the single-stranded DNA binding protein, and an extra DNA-dependent RNA polymerase. *Alteromonas* viruses vB_AspP-H4/4 and vB_AmeP_R8W have been classified into *Autographiviridae*. Phage vB_AspP-H4/4, isolated from the North Sea (34), has been used as a biological tracer in hydrological transport studies (35). Phage vB_AmeP_R8W can infect 35 *Alteromonas* strains and exhibited a strong specificity for strains isolated from deep waters (36). Phage vB_AmeM_PT11-V22 was identified as a myovirus by genomic and morphological analyses, but it lacked sequence similarity to any other known myoviruses (37). Its genome size is about 92 kb, with a very low G+C content (38%). These characteristics suggest that myovirus vB_AmeM_PT11-V22 belongs to a new type of phage, the *Myoalterovirus* genus within the *Myoviridae* family. Although the genome of bacteriophage phiAFP1 has been uploaded, it has not yet been classified into a viral family.

In this study, to gain a better understanding of marine *Alteromonas* phages, genomic, phylogenetic, and comparative genomic analyses of a novel *Alteromonas* phage, ZP6, are reported. Phylogenetic analysis based on the whole genome of phage ZP6 and comparative genomic analysis indicates that ZP6-like phages form a novel viral cluster within the *Podoviridae*. The relative abundances and distributions of *Alteromonas* phages, *Pelagibacter* phages, and cyanophages suggest that ZP6 could be prevalent in the mesopelagic zone of the temperate and tropical oceans.

## RESULTS AND DISCUSSION

### Morphology and one-step growth curve.

**FIG 1:**
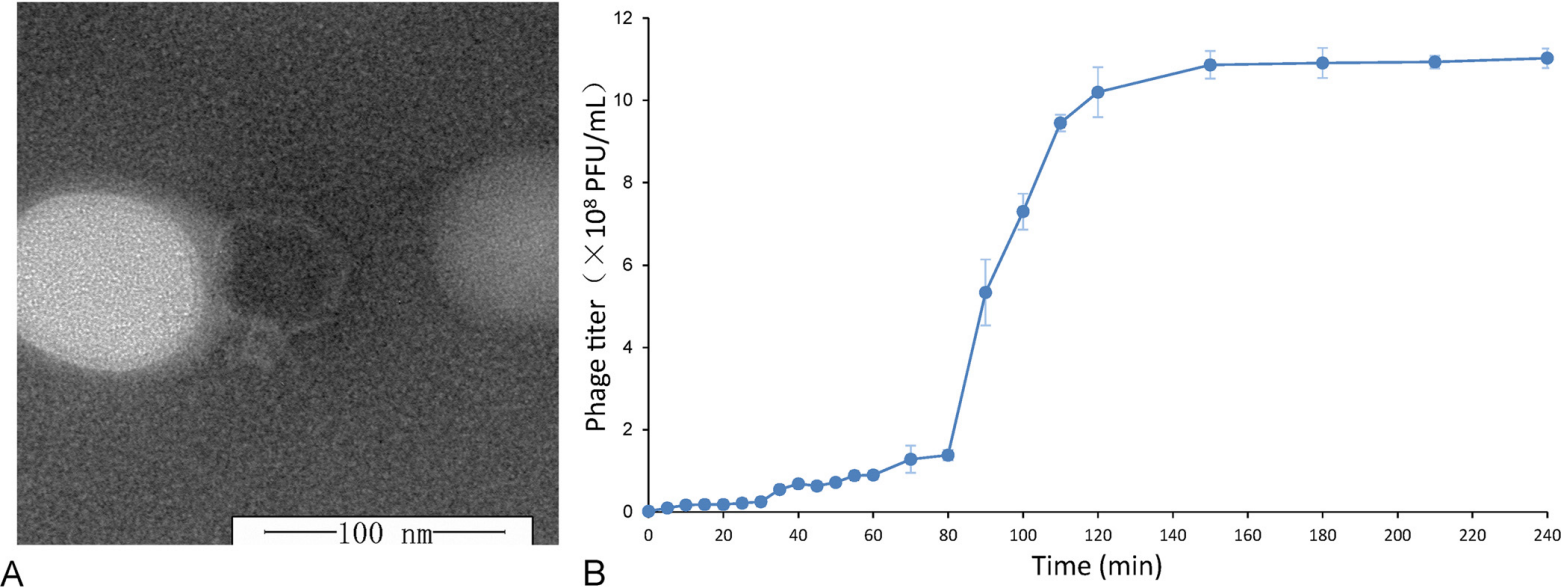
Morphology and biological properties of *Alteromonas* phage ZP6. (A) Transmission electron micrograph of ZP6. (B) One-step growth curve of *Alteromonas* ZP6. The data shown are average values from triplicate experiments, and error bars indicate standard deviations (SDs).

A marine phage, designated ZP6, that can infect the Yellow Sea variant of the Alteromonas macleodii type strain ATCC 27126 was isolated from a surface seawater sample from the coastal waters of Qingdao, Yellow Sea. Transmission electron microscopy (TEM) images showed that phage ZP6 had an isometric head (diameter of 50 to 62.5 nm [average ± standard deviation, 55 ± 3 nm]) and a short, thick tail (length of 10 to 12.5 nm [11 ± 3 nm]) (Fig. 1A) and could be classified into the *Podoviridae* family, order *Caudoviral*es. Currently, only four podoviruses of *Alteromonas* have been isolated, and these are all from coastal waters of the Mediterranean Sea (Table S1 in the supplemental material) (33). Phage ZP6 is the first podovirus of *Alteromonas* to be isolated from the west Pacific Ocean. The one-step growth curve of phage ZP6 showed that the latent period was approximately 80 min and the rise period was approximately 40 min (Fig. 1B). The burst size is close to 210 virions per cell (Fig. 1B), which is smaller than those of the other four *Alteromonas* podoviruses, vB_AmaP_AD45 P1 to P4 (500 virions per cell) (33), but larger than those of *Autographiviridae* virus vB_AmeP-R8W (88 PFU/cell) (36) and *Siphoviridae* virus R7M (182 PFU/cell) (32).

### Overall genome features.

**FIG 2:**
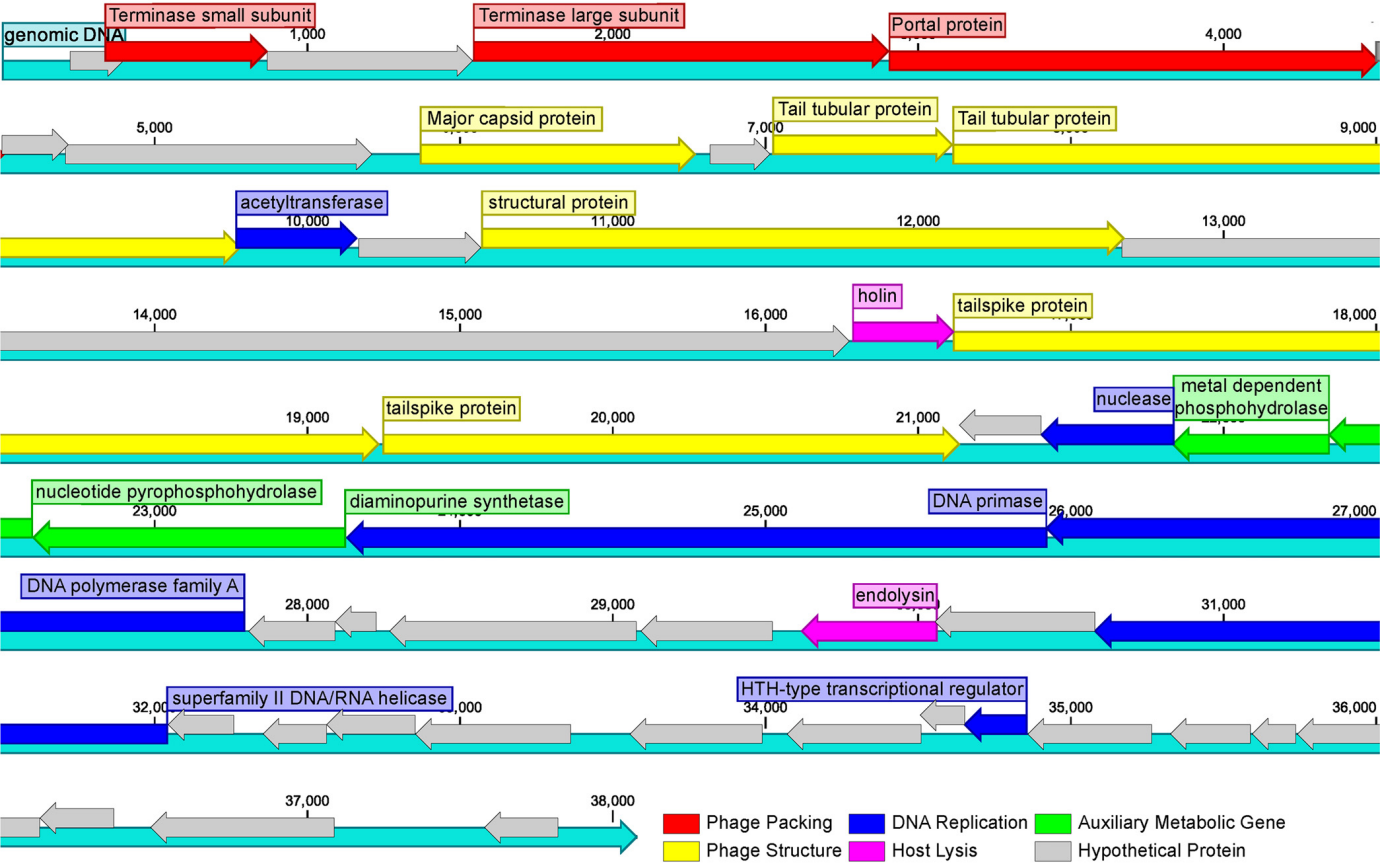
Genome map of *Alteromonas* phage ZP6. Putative functional categories were defined according to annotation and are represented by different colors. The length of each arrow represents the length of each gene.

The ZP6 genome is a linear, 38,080-bp, double-stranded DNA (dsDNA) molecule with a G+C content of 50.1% (Fig. 2); no tRNA genes are predicted. The open reading frames (ORFs) of ZP6 were identified by BLASTp, Pfam search, and HHpred analyses, and a total of 47 ORFs were predicted. Among these genes, 20 genes are predicted to have known functions (Table 1) and are grouped into three functional modules as follows: phage packaging and lysis (ORFs 2, 4, 5, 16, and 30), phage structure and assembly (ORFs 8, 10, 11, 14, 17, and 18), and DNA metabolism and replication (ORFs 12, 20, 24, 25, 32, and 40). Additionally, three auxiliary metabolic genes (AMGs) (ORFs 21, 22, and 23) were predicted, and 27 ORFs were predicted to encode hypothetical proteins.

**TABLE 1:** Genomic annotation of *Alteromonas* phage ZP6 and conserved domains detected

ORF	Position		Strand	Function	CD accession no.^*a*^	E value
	Start	Stop				
2	336	869	+	Terminase small subunit	PF03592.18 (Pfam)	4.6e−20 (HHpred)
4	1,543	2,904	+	Terminase large subunit	PF03354.17 (Pfam)	5.2e−33 (HHpred)
5	2,904	4,508	+	Portal protein	PF12236.10 (Pfam)	1.7e−55 (Hhpred)
8	5,869	6,771	+	Major capsid protein	PF19821.1 (Pfam)	5.7e−27 (HHpred)
10	7,023	7,613	+	Tail tubular protein	PF17212.5 (Pfam)	1.8e−34 (HHpred)
11	7,613	9,781	+	Tail tubular protein	6R21_f (PDB)	3.8e−78 (HHpred)
12	9,765	10,163	+	Acetyltransferase	MBT3950205.1	4e−06 (BLASTp)
14	10,570	12,675	+	Structural protein	YP_009140146.1	7e−06 (BLASTp)
16	16,285	16,617	+	Holin	PF16085.7 (Pfam)	5.1e−27 (HHpred)
17	16,614	19,235	+	Tailspike protein	5JSD_B (PDB)	7.1e−24 (HHpred)
18	19,247	21,136	+	Tailspike protein	5W6S_A (PDB)	2.4e−28 (HHpred)
20	21,839	21,402	−	Nuclease	4QBN_B (PDB)	7.5e−9 (HHpred)
21	22,347	21,832	−	Metal-dependent phosphohydrolase	PF12917.9 (Pfam)	3.4e−18 (HHpred)
22	22,601	22,344	−	Nucleotide pyrophosphohydrolase	PF03819.17 (Pfam)	2.1e−08 (Pfam)
23	23,626	22,601	−	Diaminopurine synthetase	PF00709.21 (Pfam)	2.1e−21 (Pfam)
24	25,922	23,628	−	DNA primase	PF09250.11 (Pfam)	3.3e−26 (Pfam)
25	27,796	25,919	−	DNA polymerase family A	PF09250.11 (Pfam)	1.2e−42 (Pfam)
30	30,063	29,617	−	Endolysin	YP_008051103.1	4e−30 (BLASTp)
32	32,043	30,577	−	Superfamily II DNA/RNA helicase	YP_009153058.1	2e−123 (BLASTp)
40	34,857	34,651	−	HTH-type transcriptional regulator	PF01381.22 (Pfam)	1.3e−10 (Pfam)

a Accession numbers for which the database is not named are from GenBank. CD, conserved domain.

Most of the genes associated with phage packaging are located at the beginning of the ZP6 genome. ORFs 2 and 4 encoded the terminase small (TerS) and large (TerL) subunit (38), respectively. The terminase and DNA recognition proteins mediate the packaging of dsDNA virus concatemers and require interaction of the prohead with the virus DNA (39). The TerS is thought to form a nucleoprotein structure that helps to locate the TerL at the packaging initiation site (40). ORF 5 encodes the portal protein, which controls the size of the assembled viral genome and effectively prevents the DNA from escaping from the capsid during assembly (41). ORF 16 encodes a small hydrophobic protein called holin, which oligomerizes in the cytoplasmic membrane until pores are formed. ORF 30 encodes endolysin, which cleaves the cell wall peptidoglycan. Together, they form a classical holin-endolysin lysis system. Endolysin reaches the cell wall through the pore formed by holin, degrades the host cell wall, and effectively completes the lysis. (42).

Genes related to the structure are mainly located in the middle of the ZP6 genome. ORF 8 encodes the major capsid protein (MCP), which synthesizes the protein coats of viruses that encapsulate their genetic material. ORFs 10 and 11 encode the tail tubular protein, which allows phages to inject their genomes inside the bacterial cytoplasm without disrupting the cellular integrity (43). ORF 14 was similar to ORF 17 of *Vibrio* phage phiVC8 (44), identified as a structure-related gene with unknown role. ORF 17 and 18 encode the tail spike protein, which is located at the tail of the viral particles and can help the virus deliberately identify host cells (45).

Genes related to the replication and regulation of bacteriophage DNA were mainly located in the downstream region of the ZP6 genome. ORF 12 was predicted to encode a member of the Gcn5-related *N*-acetyltransferase (GNAT) superfamily, which is a large group of evolutionarily related acetyltransferases with multiple paralogs in organisms from all kingdoms of life (31). The GNAT protein encoded by phiKMV-like viruses has the biological function of the RNA polymerase alpha subunit cleavage protein (Rac) (46). Rac can bind the β′ DNA-dependent RNA polymerase subunit, inactivate bacterial transcription, and then control the switch to late transcription (47). ORF 10 might be involved in the acetylation of histones at specific lysine residues, which is required by the process of transcriptional activation and has been implicated in chromatin assembly and DNA replication (48, 49). ORF 20 contained a virus-type replication-repair nuclease (VRR-NUC) domain. It is associated with members of the PD-(D/E)XK nuclease superfamily, such as the type III restriction modification enzymes (50). ORF 24 encodes a DNA primase, which can synthesize short oligonucleotides, usually RNA, that then act as primers to assist DNA polymerization (51). ORF 23 encodes a DNA polymerase that contains a conserved domain with PDB code 2KFZ found in Escherichia coli. (52). ORF 25 is similar to the DNA polymerase encoded by the host bacteria. ORF 32 encodes DNA helicase, which is necessary for the ATP-dependent unwinding of dsDNA, an important step in DNA replication, expression, recombination, and repair. At the same time, ORF 32 contains a conserved amino-terminal region with an SNF2 domain corresponding to the helicase-like ATP-dependent family and participating in chromatin structure remodeling (53). ORF 40 encodes a helix-turn-helix (HTH)-type transcriptional regulator that is a major structural motif capable of binding DNA. Each monomer incorporates two helices, joined by a short strand of amino acids, that bind to the major groove of DNA (54).

### Three diaminopurine genome-biosynthetic-related AMGs.

The auxiliary metabolic genes (AMGs) are phage-encoded and host-derived metabolic genes that are putatively involved in the regulation of host metabolism to increase viral replication (55, 56). Three AMGs were predicted within the ZP6 genome: ORF 21 (metal-dependent phosphohydrolase), ORF 22 (nucleotide pyrophosphohydrolase), and ORF 23 (diaminopurine synthetase).

Of these, the most interesting is diaminopurine synthetase (PurZ), encoded by ORF 23, which is involved in the replacement of adenine (A) by diaminopurine (Z) in phage genomes (57). PurZ is a homolog of adenylosuccinate synthetase (PurA) in the purine biosynthetic pathway (54). Diaminopurine (Z) can completely replace adenine (A) and form three hydrogen bonds with thymine (T). The diaminopurine genome, which is completely different from the common Watson-Crick base pairing, endows phages with evolutionary advantages for evading the attacks of host restriction enzymes (57). Homology models were constructed for amino acid sequences of the identified PurZ encoded by ORF 23 (ZpPurZ) (Table S4), PurA, and other identified PurZ proteins. The results for all PurZ proteins showed that the catalytic residue Asp^13^ (Escherichia coli PurA numbering) in PurA was without exception replaced by a Ser residue (Fig. S1). At the same time, like most PurZ-carrying phages, ZP6 contains an HD domain-containing hydrolase-like enzyme encoded by ORF 21. These HD domain enzymes exhibited dATPase activity, which can catalyze the hydrolysis of dATP to dA and triphosphate. It also catalyzes the hydrolysis of dADP and dAMP into dA, releasing pyrophosphate and phosphate (57). Therefore, the dATPase encoded by ORF 21 could facilitate Z genome synthesis by specifically removing dATP and its precursor dADP from the nucleotide pool of the host (58), preventing the incorporation of A into the phage genome. PurZ, dATPase, and DNA polymerase form the diaminopurine genome-biosynthetic system, which can evade the restriction enzyme attacks of hosts (54, 57, 59, 60). Notably, these three proteins were at similar locations in the genomes of *Vibrio* phages phiVC8, VP2, and VP5. The presence of these enzymes constitutes one of the main characteristics of the *Enhodamvirus* genus (61) and ZP6-like phages (Fig. S4).

ORF 22 encodes nucleotide pyrophosphohydrolase (MazG). MazG enables the host cells to stop programmed cell death by hydrolyzing (p)ppGpp and improves the survival rate of the host in nutrient-depleted environments (62, 63). The role MazG plays in host stringer responses enables it to be classified as a class I AMG (56). More interestingly, a recent study has suggested that MazG shows a preference for dGTP and dCTP as substrates, suggesting a role in recycling host nucleotides (64). Associated with the recently published diaminopurine DNA genome, the MazG carried by ZP6 may provide dGMP as a substrate for PurZ and, thus, improve the level of dZTP (57).

### Phylogenetic and comparative genomic analyses.

**FIG 3:**
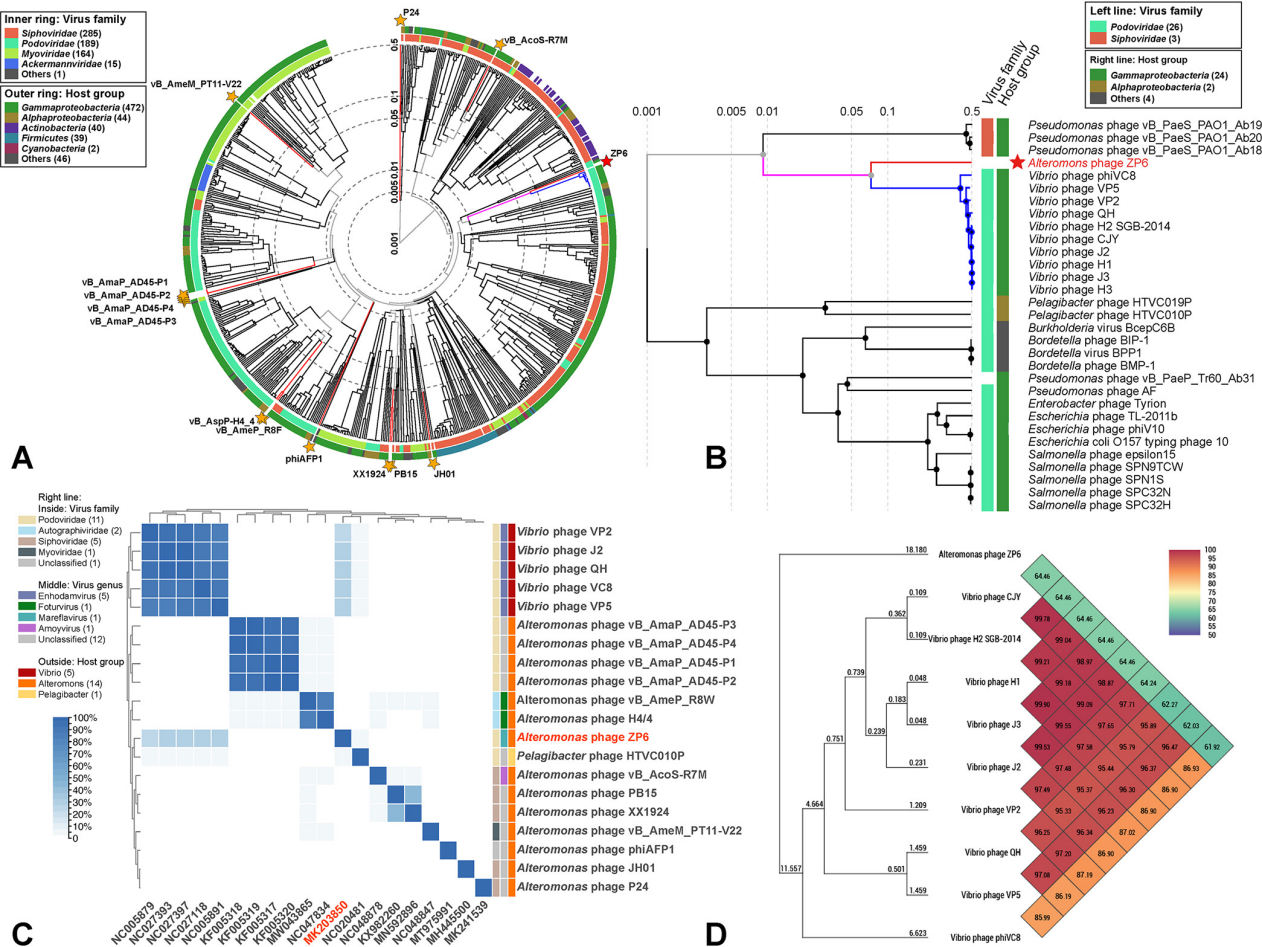
Phylogenetic and comparative genomic analyses of *Alteromonas* phage ZP6. (A) Phylogenetic tree of all 14 *Alteromonas* phages and 100 selected viruses most closely related to *Alteromonas* phages, constructed by using VipTree. The colored rings represent the virus families (inner ring) and host groups (outer ring). These trees are calculated by BIONJ according to the genome distance matrix and take the midpoint as the root. (B) Phylogenetic tree of *Alteromonas* phage ZP6 and the 30 closest virus genomes. (C) The heat map shows shared genes among *Alteromonas* phage ZP6, other *Alteromonas* phages, typical enhodamviruses, and *Pelagibacter* phage HTVC010P. The ratio of shared genes was based on all-vs-all BLASTp analysis, which was performed by using OrthoFinder with the following parameters: cutoff E value, <1e−10; identity, >30%; and alignment region covering >50% of the shorter sequence. The cluster method was complete, which defined the class-to-class distance as the complete distance between samples. The numbers in parentheses in the keys of panels A, B, and C represented the number of different classification of viruses. (D) Heat map of OrthoANI values of ZP6 and typical enhodamviruses. The values were calculated by using OAT software.

Since the known *Alteromonas* phages are rare and distributed in different phage families, it is difficult to carry out a comparative genomics analysis within the *Alteromonas* phage group (Table S1). Therefore, 2,687 phage genomes were used as reference sequences to construct phylogenetic trees using VipTree (https://www.genome.jp/viptree) based on the whole-genome amino acid sequences of phage ZP6 and other *Alteromonas* phages (65). tBLASTx and VipTree were used to perform the genome comparisons, in order to describe the relationship between phage ZP6 and its closest relatives. One hundred viruses related to *Alteromonas* phages were selected to clearly display the evolutionary relationship between *Alteromonas* phages and related phages (Fig. 3A). Preliminary observation of the phylogenetic trees was consistent with the morphological characteristics of ZP6 and its host. The results indicate that ZP6 is closely related to podoviruses and *Gammaproteobacteria* phages. This suggests that ZP6 is a divergent podovirus within the *Podoviridae* family. The close relationship between ZP6 and several *Podoviridae* was in accordance with the results of the morphological analysis. Additionally, 30 closely related genome sequences of ZP6 were selected to draw a rectangular proteomic tree (Fig. 3B). The ZP6 genome was grouped with some *Vibrio* phages, which all belong to *Enhodamvirus* (according to the virus taxonomy of ICTV, *Enhodamvirus* is a genus of *Podoviridae*). This group of phages encode proteins that are poorly related to any other phage proteins and form a separate branch, far from the other sequences, and represent a novel viral cluster. Four protein phylogenetic trees were constructed using hallmark conserved viral proteins, including the TerL, MCP, DNA polymerase, and portal protein (Fig. S2). The results are consistent with the proteomic tree generated by VipTree (Fig. 3). Although ZP6 is weakly clustered with *Enhodamvirus* in the phylogenetic trees of marker genes and the complete genome, the results still clearly demonstrate that ZP6 forms a new single clade by itself.

All-vs-all BLASTp analysis was used to study the phylogenetic relationships between ZP6 and other cultured *Podoviridae* phages (336 podovirus genomes from NCBI RefSeq database) (Fig. S3) and further verify the unique status of ZP6. The results show that only enhodamviruses and *Pelagibacter* phage HTVC010P have genes homologous with those of ZP6 (threshold values: E value, <1e−5; identity, >30; and alignment coverage per query [qcov], >50%). The ratio of shared genes between ZP6 and enhodamviruses (Fig. 3C) is less than 30% (∼27.65% to 29.47%). *Pelagibacter* phage HTVC010P (66) has three conserved genes homologous with those of ZP6 (*mcp*, *TerL*, and portal protein gene). The average nucleotide identity (ANI) values between ZP6 and the nine typical enhodamviruses that are most closely related to ZP6 in the phylogenetic tree were calculated by OrthoANI (ANI by orthology) (Fig. 3D). The ANI values between ZP6 and the other nine enhodamviruses ranged from 61.92 to 64.46, which is far lower than the ANI values among enhodamviruses (85.99 to 99.90). Phages are regarded by the ICTV as being members of the same genus when their nucleotide sequence identities are greater than 70% (67). All phylogenetic analyses illustrate that ZP6 is significantly different from all other isolated phages and should be classified as a representative of an undiscovered viral group.

**FIG 4:**
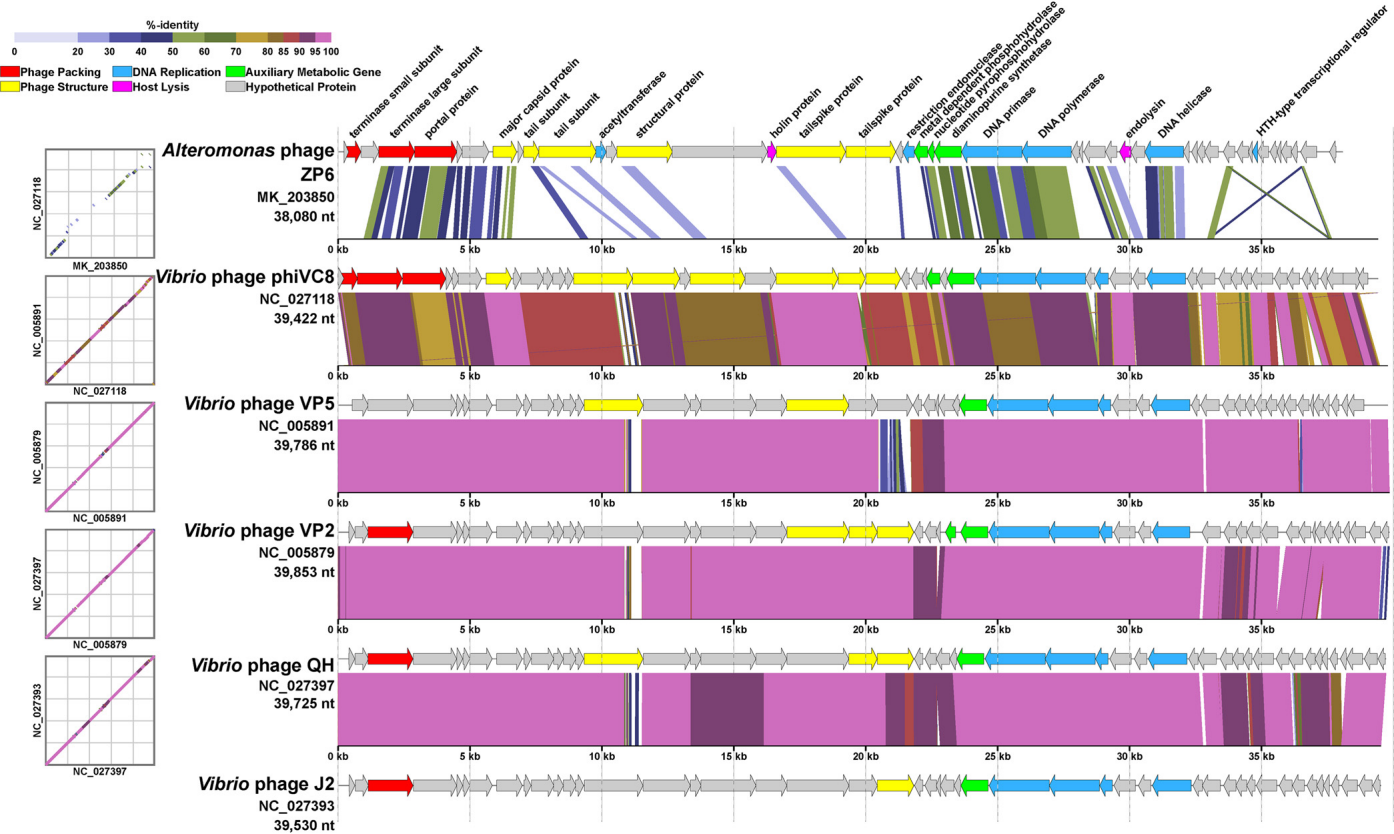
Genomic comparisons between *Alteromonas* phage ZP6 and typical enhodamviruses. The predicted functions of proteins are indicated by different colors of arrows representing genes. The shading below each genome indicates sequence similarities between the genomes, with different colors representing the levels of similarity.

The results of a comparative genomic analysis of ZP6 and typical enhodamviruses (Fig. 4), i.e., *Vibrio* phage phiVC8 (GenBank accession number NC_027118) (44), *Vibrio* phage VP5 (NC_005891) (61), *Vibrio* phage VP2 (NC_005879), *Vibrio* phage QH (NC_027397), and *Vibrio* phage J2 (NC_027393), by BLASTx (68) verified the conclusion that ZP6 belongs to a previously unreported genus. The gene architecture of the five enhodamviruses showed that the arrangement of functional genes is relatively conservative, with homologous genes being arranged in the same order. However, unlike enhodamviruses, in the ZP6 genome, taking those of ZP6 and *Vibrio* phage phiVC8 as examples, only the genes encoding the packaging module TerL (amino acid identity of 32.96%, calculated by BLASTp), the portal protein (45.04%), the DNA replication module DNA primase (45.1%), DNA polymerase (52.61%), superfamily II DNA/RNA helicase (43.24%), and the iconic AMG of enhodamviruses, PurZ (52.61%), reflect this pattern. Among the structural modules, *mcp* (37.54%) and the major tail subunit gene (30.1%) were homologous to those of enhodamviruses. The main difference between ZP6 and enhodamviruses is the module related to tail structure and host lysis. ZP6 contained four tail protein domains and had no similarity with enhodamviruses, which may be the main reason for their infection of different hosts (43, 45). On the other hand, enhodamviruses do not contain a gene related to host lysis, but ZP6 contains a complete lysis system.

**FIG 5:**
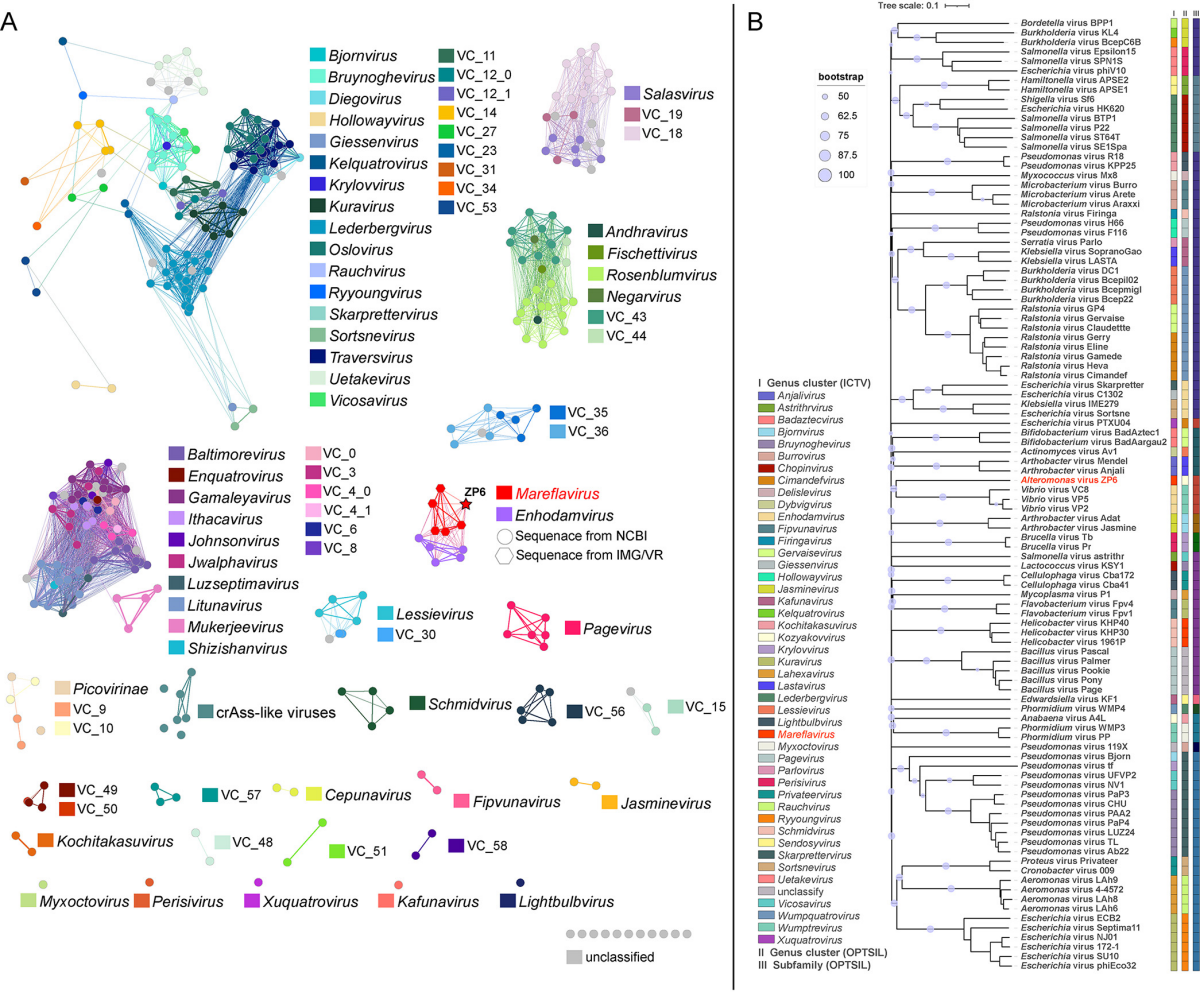
(A) Gene content-based viral networks showing all of the *Podoviridae* viruses from the NCBI RefSeq database and five environmental viruses related to ZP6 from IMG/VR. The nodes represent the viral genomic sequences. The edges represent the similarity scores between genomes based on shared gene content. The isolated viral sequences are indicated by filled circles, and environmental viral sequences are indicated by regular hexagons. Among those, the star represents *Alteromonas* phage ZP6. Viral genomes that belong to different viral clusters are indicated by different colors. (B) Whole-genome-based phylogenetic tree constructed by VICTOR with the formula d6. ICTV and OPTSIL clusters are at the genus and family levels. Each genus is indicated by a unique color. ZP6 is shown in red. Bootstrap values of ≥50 are shown.

Although ZP6 apparently has characteristics belonging to a new genus, it has been difficult to characterize this new genus with certainty from only a single phage. So, five metagenome-assembled viral genomes (MAGs) with high homology (shared genes, ∼52.38% to 53.65%) to ZP6 were mined from Integrated Microbial Genome/Virus (IMG/VR) 3.0 data sets (Tables S2 and S3). Comparative genomic analysis of the six ZP6-like viruses and enhodamviruses (Fig. S4) showed that the ZP6-like viruses contained eight core genes that were different from those of the enhodamviruses. Four of them (ORFs 2, 10, 11, and 14 of ZP6) were functional protein genes coding for terminase small subunit, major tail subunit, structural protein, and restriction endonuclease, respectively. Then, 343 viruses (336 from NCBI RefSeq, ZP6, and 6 ZP6-like viruses) were divided into genus or subfamily level groups using vConTACT 2.0 (Fig. S5) (67). Using this method, 60 virus clusters were identified in the whole data set. ZP6 overlapped with VC_40 (*Enhodamvirus*) and VC_39 (viral operational taxonomic unit [vOTU] of IMG/VR [Sg_132140]) but had no correlations with other phages (Fig. 5A). At the same time, whole-genome-based phylogenic analysis was conducted for ZP6 and the other 45 different genera of *Podoviridae* (Fig. 5B). The whole-genome phylogeny and OPTSIL taxon results all implied that ZP6 did not belong to any of the identified genera. When this is combined with the network analysis and comparative genomics analysis, it is clear that ZP6 should be classified as a new genus, here named *Mareflavirus.*

### Distribution in marine environments.

**FIG 6:**
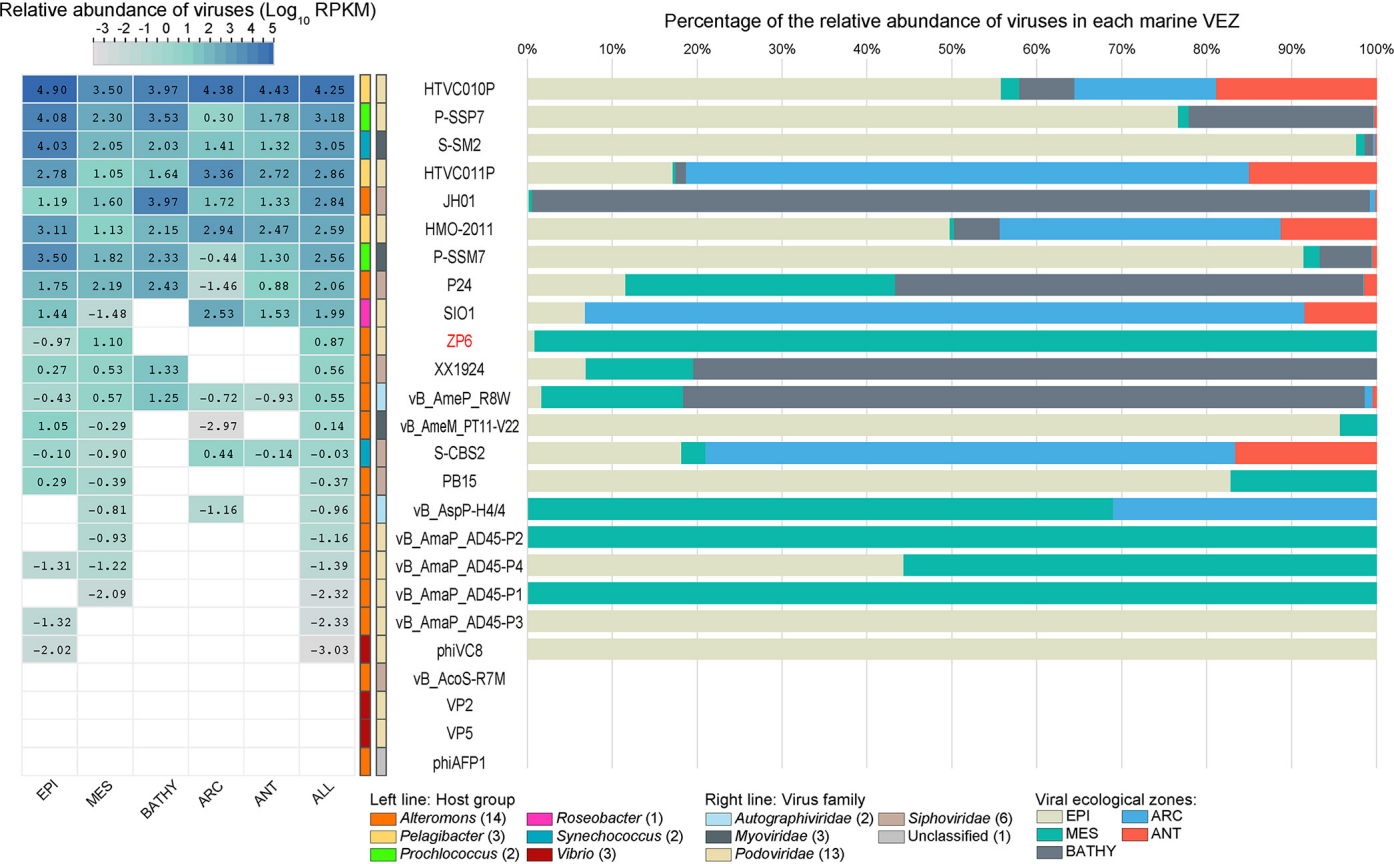
Relative abundances of *Alteromonas* phage ZP6 compared to the abundances of representative *Pelagibacter* phages, cyanophages, typical enhodamviruses, and other *Alteromonas* phages in the 154 viromes of the Global Ocean Viromes data set (GOV 2.0). Relative abundances, expressed by RPKM (reads per kilobase per million mapped reads) values, were calculated using the metagenomics tool minimap2. Left, relative abundances of different bacteriophages in five marine viral ecological zones (VEZs) defined by the GOV2.0. Values were normalized by the number of databases of each VEZ, and results were log_10_ transformed for description. Right, distributions of phages in five VEZs, shown as percentages. ARC, Arctic; ANT, Antarctic; BATHY, bathypelagic; EPI, temperate and tropical epipelagic; MES, temperate and tropical mesopelagic.

The biogeographical distribution of *Alteromonas* phage ZP6 was characterized in 154 viral metagenomes from five viral ecological zones (VEZs) of the Global Ocean Viromes (GOV2.0) data set: Arctic (ARC), Antarctic (ANT), bathypelagic (BATHY), temperate and tropical epipelagic (EPI), and temperate and tropical mesopelagic (MES). After being normalized by the number of databases of each ecological environment, the relative abundances of the viral genomes were log_10_ transformed based on 10 reads per kilobase per million (RPKM) (Fig. 6).

These results confirmed the high abundances of pelagiphages, the SAR116 bacterial-clade-infecting phage HMO-2011, and cyanophages, as shown in previous studies from Pacific, Indian, and Global Ocean viromes (11, 66, 69). Most of the *Alteromonas* phages were less abundant than pelagiphages, cyanophages, and HMO-2011, except for *Alteromonas* siphophage JH01, which had a higher abundance than HMO-2011 and was comparable to cyanophages (Fig. 6). *Alteromonas* phages were detected in five different VEZs, which is in accordance with the widespread distribution of their hosts (13, 14). Based on the relative abundances in the GOV 2.0 database, *Alteromonas* siphophages (JH01, P24, and XX1924) and autographivirus vB_AmeP_R8W were relatively abundant, had similar distribution patterns, and were abundant in the BATHY VEZ. Four *Alteromonas* podophages (ZP6, vB_AmaP_AD45-P2, vB_AmaP_AD45-P4, and vB_AmaP_AD45-P1) and one *Autographiviridae* phage, vB_AspP-H4/4, had similar distribution patterns and were mainly detected in the MES VEZ, which was consistent with the distribution of their hosts (70, 71).

### Conclusion.

Culturing viruses infecting major components of bacterial assemblages is likely to provide important insights into novel viral sequences from metagenomic data sets. Considering the ecological significance of *Alteromonas*, the research on its bacteriophages is still at an elementary level. In this study, we describe a novel *Alteromonas* phage, ZP6, with unique genomic characteristics and phylogenetic position. ZP6 contains a diaminopurine genome-biosynthetic system, which could help it evade the attack of host restriction enzymes. ZP6 represents a new viral genus of podovirus, namely, *Mareflavirus.* The establishment of *Mareflavirus* will undoubtedly contribute to our knowledge of the little-known *Alteromonas* phages, deepen our understanding of the physiology, genetic diversity, and genomic characteristics of phages in different aquatic environments, provide a novel phage-host system for interaction analysis, and contribute to the data mining of the massive metagenomic data set.

## MATERIALS AND METHODS

### Location and sampling.

Surface seawater samples (50 liters per seawater sample) from the coastal waters off Qingdao in the Yellow Sea (120°19′23″E, 36°4′4″N) were collected on 22 July 2018. The water samples were processed immediately after collection. A subsample of the water sample was prefiltered using 3-μm-pore-size filters to remove the larger plankton and particles, followed by filtration through 0.2-μm-pore-size, low-protein-binding polyvinylidene difluoride (PVDF) filters (Millipore) to remove any remaining bacteria and phytoplankton. Using tangential flow filtration (laboratory scale, 50 kDa; Millipore), the virus-containing seawater was concentrated 500-fold to give a 100-ml sample. The samples and the original seawater were stored at 4°C in the dark until experimentation (72).

### Bacterial strain isolation and identification.

Using serial dilution, a host bacterial strain was isolated from the unfiltered seawater sample and then incubated in liquid Zobell medium at 28°C (73, 74). For molecular identification, the 16S rRNA gene was amplified by PCR. The result was analyzed via 16S rRNA gene sequence analysis (see the supplemental material) (72), and a BLASTn search of the 16S rRNA gene sequence was performed. The 16S rRNA gene sequence of the host bacterial strain of phage ZP6 had a 99% similarity to Alteromonas macleodii type strain ATCC 27126 (accession number CP003841).

### Phage isolation and purification.

The phage was isolated from the same seawater sample after filtering a subsample through a 0.22-μm Millipore membrane to remove the bacteria and phytoplankton. The isolation of phage plaques was by gradient dilution and the double-layer agar plate method (75), followed by using the soft-agar overlay method for plaque analysis (76). Briefly, 1 ml of the filtrate and 0.2 ml of indicator bacteria were placed into 5 ml of soft, warm agar (0.6%), agitated, and then poured onto petri dishes to form plaques. Phages were purified by picking a single plaque, suspending it in SM buffer (100 mM NaCl, 8 mM MgSO_4_, 50 mM Tris HCl [pH 7.5]), and then incubating it for 1 h at 37°C. The purification step was repeated three times, and then the purified phages were amplified and stored at 4°C.

### Morphology study by TEM.

The purified phage samples were negatively stained with phosphotungstic acid (2%, wt/vol, pH 7.2). Transmission electron microscopy (TEM) (JEOL JEM-1200EX; JEOL, Japan) at 100 kV was used to provide images of phage ZP6 purified particles (77). The phage was examined at a magnification of ×400,000. Phage size was calculated from the electron micrographs (78).

### One-step growth curve.

A one-step growth curve was used to determine the burst size (the average number of phage particles that a single infected bacterium can produce) of phage ZP6. The latent period of the phage was determined by the double-layer agar plate method (79). The latent period is defined as the time interval between absorption and the beginning of the first burst. The burst size was calculated as the ratio of the final number of phage particles to the initial number of infected host cells at the beginning of the test (80). The bacterial culture in exponential growth phase (2 × 10^8^ CFU/ml) was mixed with 1 ml of the phage to produce a multiplicity of infection (MOI) of 0.1 (adsorption at 25°C). The unabsorbed phage was removed by centrifugation (12,800 × *g* for 30 s). Samples were then taken every 5 min for 1 h, followed by sampling every 10 min for the next hour (81). The last sample was taken half an hour after that. This experiment was repeated three times (78). After collection, a double-layer plate was coated with the samples and cultured overnight. The numbers of phage plaques were counted to calculate the titers of the phage in different periods to determine the growth states of the phage.

### Genome sequencing and bioinformatics analysis.

The DNA of phage ZP6 was extracted according to the experimental protocol used by Verheust et al. (82). The extracted phage DNA was sequenced using the Illumina Miseq 2 × 300 paired-end sequence method. The gaps between remaining contigs were closed using GapCloser and GapFiller, with purified genomic DNA as the template. The termini were identified by using PhageTerm (83, 84). The reads with the maximum coverage were considered phage termini (see the supplemental material). Genome annotations were analyzed using RAST (http://rast.nmpdr.org/). Nucleotide sequences and protein sequences were scanned for homologs using BLAST (http://blast.ncbi.nlm.nih.gov/, database updated on 25 June 2021), a Pfam search with default parameters (https://pfam.xfam.org/search/sequence), and an HHpred search carried out using the online server (https://toolkit.tuebingen.mpg.de/hhpred) (73, 76, 85–89). The tRNAscan-SE program was used to predict tRNA sequences (https://lowelab.ucsc.edu/tRNAscan-SE/) (90). Genome mapping was performed using CLC Main Workbench 20.

### Phylogenetic analysis and comparative genomic analyses.

The proteomic tree, based on the whole-genome amino acid sequences of phage ZP6 and *Alteromonas* phages, was generated using VipTree (https://www.genome.jp/viptree) (65). tBLASTx and VipTree were used to perform the genome comparisons, in order to describe the relationships between phage ZP6 and its closest relatives.

Phylogenetic trees of viral conserved proteins (MCP, TerL, DNA polymerase, and portal protein) were constructed to evaluate the evolutionary relationships among ZP6 and other diverse phages. Sequence alignments were constructed with MUSCLE (91), evaluated for optimal amino acid substitution models, and run with IQtree 2.0 (92) with a bootstrap of 1,000.

All-vs-all BLASTp analysis was performed by using OrthoFinder (93, 94) to calculate the percentage of shared genes between phage ZP6 and all complete podovirus genomes from the NCBI RefSeq database.

vConTACT 2.0 (95, 96) performs guilt-by-contig-association classification based on the ICTV taxonomy data set to cluster and provide taxonomic context for the sequencing data. In order to describe the taxonomic information of ZP6 in detail, the phage group was expanded using BLASTp. To search for homologous phages with more than 50% shared genes with ZP6, each coding sequence of ZP6 was queried against the Integrated Microbial Genome/Virus (IMG/VR) database (10, 97, 98) (E value, <1e−5; identity, >30; and alignment region covering >50%) (Tables S2 and S3). The selected sequences were compared with ZP6 as a group in the vConTACT analysis to obtain more accurate results (similar sequences were selected by Diamond, and all satisfied the following parameters: E value, <1e−5; alignment region covering more than 50% of the shorter sequence; and identity >30%) (99). The edge-weighted model network based on vConTACT analysis was exhibited by Gephi (100).

Virus Classification and Tree Building Online Resource (VICTOR; https://ggdc.dsmz.de/victor.php) (101) was used to determine the taxonomic position of ZP6 in the podoviruses. Ninety-nine podoviruses from 45 different genera were selected from the ICTV taxonomy releases to construct a phylogenetic tree with ZP6. The result was visualized with iTol (version 5) (102). All pairwise comparisons of the nucleotide sequences were conducted using the Genome-BLAST Distance Phylogeny (GBDP) method under settings recommended for prokaryotic viruses (101, 103). Taxon boundaries at the species, genus, and family levels were estimated with the OPTSIL program, using the recommended clustering thresholds and an F value (fraction of links required for cluster fusion) of 0.5 (101, 103, 104).

OrthoANI (average nucleotide identity by orthology) (105) was obtained using the orthogonal method to determine the overall similarity between two genomic sequences.

### Ecological distribution in the ocean.

The relative abundances of viral genomes in the Global Ocean Viromes 2.0 (GOV 2.0) database (9), expressed by RPKM (reads per kilobase per million mapped reads) values, were calculated using the metagenomics tool minimap2 (parameters: –min-read-percent-identity 0.95 –min-read-aligned-percent 0.75 -m rpkm) (106). GOV 2.0 divided the 154 virome databases into five viral ecological zones (VEZs), including the Arctic (ARC), Antarctic (ANT), bathypelagic (BATHY), temperate and tropical epipelagic (EPI), and temperate and tropical mesopelagic (MES). The relative abundances of ZP6 in the five VEZs were analyzed to study its global oceanic distribution. Meanwhile, the relative abundances of ZP6 were compared with those of pelagiphage HTVC010P, phage HMO-2011, marine cyanophages P-SSP7 and P-SSM7, and roseophage SIO1, all of have widespread distributions in the ocean (11, 66), and other *Alteromonas* phages and some *Vibrio* phages that have a taxonomic association with ZP6.

### Data availability.

The complete genome of bacteriophage ZP6 has been deposited in NCBI under accession number MK203850.
