# Supplementary material for: Genome and Ecology of a Novel Alteromonas Podovirus, ZP6, Representing a New Viral Genus, Mareflavirus
Source: Microbiol Spectr. 2021 Oct 13;9(2):e00463-21. doi: 10.1128/Spectrum.00463-21 (PMC8515928; doi:10.1128/Spectrum.00463-21)
Supplement: Supplemental file 1 — Supplemental material. Download SPECTRUM00463-21_Supp_1_seq11.pdf, PDF file, 4.0 MB [file spectrum00463-21_supp_1_seq11.pdf]

Supplemental materials

Supplementary Material 1. 16S rRNA gene sequence of host.

Supplementary Material 2. The termini sequence of *Alteromonas* phage ZP6.

Supplementary Material 3. Amino acid sequences of identified PurZ encoded.

Fig. S1. Multiple sequence alignment of six different diaminopurine synthetases (PurZs) and EcPurA. dGMP, ATP and Asp binding residues are highlighted in magenta, cyan and yellow colors, respectively. Positions of mutations on PurZs were indicated by red boxes. Abbreviations: *SbPurZ, Sinobacteraceae bacterium*; *SpPurZ, Salmonella* phage PMBT28; *ApPurZ*, *Acinetobacter* phage SH-Ab 15497; *VpPurZ, Vibrio* phage phiVC8; *ZpPurZ, Alteromonas* phage ZP6; *CpPurZ*, cyanophage S-2L; *EcPurA, Escherichia coli* PurA.

Fig. S2 Maximum likelihood phylogenetic tree based on the amino-acid sequences of terminase large subunit (TerL), major capsid protein (MCP), DNA polymerase and portal protein with 1,000 replicates. *Alteromonas* phage ZP6 was highlighted in red and *Enhodamviruses* were labelled in blue.

Fig. S3 Heatmap of shared genes between *Alteromonas* phage ZP6 and 336 prodovirus genomes from NCBI RefSeq database. The ratio of shared genes was based on all-to-all BLASTP analysis, with a cutoff E-value < $1e^{-10}$, identity more than 30% and the alignment region covered more than 50% of the shorter sequence. Cluster method was complete.

Fig. S4. Genomic comparisons of *Mareflavirus* with typical *Enhodamviruses*. The shading indicates sequence similarities between the genomes, different colors represent the level of similarity. The predicted functions of proteins are indicated by different colors. Different border colors represent genes shared by *Mareflavirus* and *Enhodamvirus* or only appear in *Mareflavirus*.

Supplementary Material 1

>16S RNA of host

GGCAGCCAGCCTAACCCATCCAAGTCGAATGGAACATTTCTAGCTTGCTAGAAGATGGCGA
GTGGCGGAAGGGTGAGTAATTCTTGGGAACTTGCCTTTTCGAAGGGGATAACAGTTGGAAA
CGCCTGCTAATACCGCATAATGTCTTCGGACCAAACGGGGCTTCGGCTCCGGCGCAAAGAG
AGGCCCAAGTGAGATTAGCTAGTTGGTAAGGTAACGGCTTACCAAGGCGACGATCTCTAGC
TGTTCTGAGAGGAAGATCAGCCACACTGGGACTGAGACACGGCCCAGACTCCTACGGGAG
GCAGCAGTGGGGAATATTGCACAATGGGGGAAACCCTGATGCAGCCATGCCGCGTGTGTGA
AGAAGGCCTTCGGGTTGTAAAGCACTTTCAGTTGTGAGGAAAAGTTAGTAGTTAATACCTG
CTAGCCGTGACGTTAACAACAGAAGAAGCACCGGCTAACTCCGTGCCAGCAGCCGCGGTA
ATACGGAGGGTGCGAGCGTTAATCGGAATTACTGGGCGTAAAGCGCACGCAGGCGGTTTGT
TAAGCTAGATGTGAAAGCCCCGGGCTCAACCTGGGATGGTCATTTAGAACTGGCAGACTAG
AGTCTTGGAGAGGGGAGTGGAATTCCAGGTGTAGCGGTGAAATGCGTAGATATCTGGAGGA
ACATCAGTGGCGAAGGCGACTCCCTGGCCAAAGACTGACGCTCATGTGCGAAAGTGTGGG
TAGCGAACAGGATTAGATACCCTGGTAGTCCACACCGTAAACGCTGTCTACTAGCTGTGTGT
GCCTTTAAGGCTGTGCGTAGCGAAGCTAACGCGATAAGTAGACCGCCTGGGGAGTACGGCC
GCAAGGTTAAAACTCAAATGAATTGACGGGGGCCCGCACAAGCGGTGGAGCATGTGGTTT
AATTCGATGCAACGCGAAGAACCTTACCTACACTTGACATGCTGAGAAGTTACTAGAGATA
GTTTCGTGCCTTCGGGAACTCAGACACAGGTGCTGCATGGCTGTCGTCAGCTCGTGTCGTG
AGATGTTGGGTTAAGTCCCGCAACGAGCGCAACCCTTGTCCTTAGTTGCCAGCCTTAAGTT
GGGCACTCTAAGGAGACTGCCGGTGACAAACCGGAGGAAGGTGGGGACGACGTCAAGTC
ATCATGGCCCTTACGTGTAGGGCTACACACGTGCTACAATGGCATTTACAGAGGGAAGCGA
GACAGTGATGTGGAGCGGACCCCTTAAAGAATGTCGTAGTCCGGATTGGAGTCTGCAACTC
GACTCCATGAAGTCGGAATCGCTAGTAATCGCAGGTCAGAATACTGCGGTGAATACGTTCCC
GGGCCTTGTACACACCGCCCGTCACACCATGGGAGTGGGATGCAAAAGAAGTAGTTAGTCT
AACCTTCGGGAGGACGA

Supplementary Material 2

>ZP6 terminal repeats sequence

CGGCCTCTGGGCGGCCCGTTCGTGGACCGCGGCGGACCGATGTGCAGCCATGCTGTATATG
TTCCACGGCACTTGACCCATGTTCCACGGGCCTTTCGGGCCTCTCTCGATAGAGCACACTC
GAGGCCCCGTGTCAACCCCTAGCAATCGCCCAGCCACGCTTCAAGTTCCTCCAGTGCTTGT
TCTGGTGCAATACCGTCTGCAACGCGTTCCTCGTACCAAGCCTCGGCCTGTTCGCGGTACTC
GCTCGGTGCGTCCTGTAACACGCTCTCAAGTGTTGACCCCTGCGCCAGCCCCAGTAAACCG
TCCAGTGCGCGGCCTGCGTTGCCCAAAACAGGTGCGTTGTCTAGTGCTTCGTTTAGTGCGT
TTCGTGCGTCGTTGTTCATCTTCGTTCTCCTGCCCGTCGTTCGGGGCGTTTGCGTCTCGGCC
CTGTTGCCTCGACTGCCCCTGCATTGTAACACGCTCGGCTGCTCGTGTCAAGTGCTCGCTGC
AGCAGGCGGGGGACAAAAACGCGTACGGGAACGCGACACACGCGCGCAACACGCGCACG
GGGAAAGGACCGCGCAACACGGGTGTCCATGCGCGCAAATACGCGTATACGGGTACACGC
CGCGCGAACGCGGGTCCCCGAGCCAACTTCACC

Supplementary Material 3

>TXG97710.1 hypothetical protein E6R08_06220 [Sinobacteraceae bacterium]
MLKKARLIIDLQFGSTGKGLIAGYLAARHNPDTLITAWAANAGHTYIDSEGTKFVHTMLANGI
VSKNLRRILIGPGSLINPDALVAEINEALALGVDLSNVKLVIHPHAAVIQQRHIEEEQGPMTKIGS
TKKGVGAAAIHRIRRDPDDNNVAVNALQGHFLGQYVGTIEEYNEQLNAAEIAQIEGAQGYSLS
MYHGMYPYTTSRDVTTAQIFADCGLPISWLARTEVIGTVRTFPIRVANRFDEQGTQIGYSGGCY
PDQHEMAWPELGLEAELTTVTKLPRRIFSLSFIQLYEAMRINGVNKLFVNFANYLRTPGEYRRV
MDGIQAVAVGSGADIGWIGFGPTDDHVIETPPELVTANLHSDRLMALWQSTNVTGGKMPVPAQ
HADFGQESLA

>AUZ95522.1 adenylosuccinate synthetase [Salmonella phage PMBT28]
MNSNKKATIVVDAQFGSTGKGLIAGYLAERDQPDVVMTAWSANAGHTYINAEGRKFVHCML
ANGIVSPKLTTVLIGPGSQMNAELLRDEILSCADLLQGKTILLHASAALILQKHVEEEAGPMTKI
GSTKKGCGAAMIAKIRRNPDDNNTVGANGDYMEEHIYGPVREAGVFIRTATNAEYMAVVYDA
ERIQVEGAQGFSLGINNGFYPYVTSRECTPAQVAVDVNLPLAFIDKVVACMRTLPIRVANRYND
KGEQIGWSGPCYPDQKELDWEKDLGMEAELTTVTKLPRRIFTFSYEQTKAALEVIRPDEVFLN
FCNYLEPDEVAAVIGTIERAAHELKVPGPMPLARYYGYGPTVNDIDLDMRHQ

>AUG85481.1 adenylosuccinate synthetase [Acinetobacter phage SH-Ab 15497]
MKKATVICDMQFGSTGKGLIAGFLAERDQPDVVVTAWSANAGHTYINREGRKWVHCMLANG
IVSPKLKAVLIGGGSQMSIPTLISEIMGSLDILQGKSILIHENACIIQQRHVEEEAGPMTKIGSTKK
GCGAAMMEKIRRNPESKIVAKDFIDDGLEIPDFKLDGTVGFKDISRHFEELGVCIKVVSNEVYL
AVLHKAERVQVEGAQGFSLGLHNGFYPYVTSRECTPAQICSDCNVPISMVDKVVGTMRTYPIR
VANRFDDEGKMVGWSGPCYSDQTELTWEQMGVTPEKTTVTKLTRRIFSFSRMQTRQAMLVC
MPDEIFLNFANYCASEDELASIIEVISNEGGDVSYIGWGDSAAHIETTLEGDWSDDTNPLFNQY
NKSSNIA

>YP_009140156.1 adenylosuccinate synthetase [Vibrio phage phiVC8]
MKNVDLVIDLQFGSTGKGLIAGYLAEKNGYDTVINANMPNAGHTYINAEGRKWMHKVLPNG
IVSPNLKRVMLGAGSVFSINRLMEEIEMSKDLLHDKVAILIHPMATVLDEEAHKKAEVGIATSIG
STGQGSMAAMVEKLQRDPTNNTIVARDVAQYDGRIAQYVCTVEEWDMALMASERILAEGAQ
GFSLSLNQEFYPYCTSRDCTPARFLADMGIPLPMLNKVIGTARCHPIRVGGTSGGHYPDQEELT
WEQLGQVPELTTVTKKVRRVFSFSFIQMQKAMWTCQPDEVFLNFCNYLSPMGWQDIVHQIEV
AAQSRYCDAEVKYLGFGPTFNDVELREDVM

>AZS06526.1 adenylosuccinate synthetase [Alteromonas phage ZP6]
MNVKMIVDLQFGSTGKGLIAGYLAERDQPDCVVNANMPNAGHTYINDEGRTWIHKVLPNGIV
SPKLKYVMIGPQSVFSLEQLRKEYKESFDLMSKVGLIIHESAVVLQDIHREVEQQALSGISSTM
QGSAAATVEKMMRNPNRDSRAKTVLKGTEFEECVTDHRAYQTIMQGCDRIQAEAAQGYSLG
LNAGFWPYCTSRDCTPQRFLADMGIPLLNLDLHVVGTARVHPIRVGNTPDGYSGDWYPDQEEI
TWESIGQKAELTTVTKRARRIATWSWLQITDALNDCAPDEIFLNFCNYDTDQAVDIASKLPEIA
YMGYGPARQDIAEVANHGGQITVLGQI

>CpPurZ_359aa_translated_from_SRA_SRR8295598_resequenced_in_2018_correct
MLSIPPYYRVKNCNLIVDCQYGSTGKGLLAGYLGALEAPQVLCMAPSPNAGHTLVEEDGTAR
VHKMLPLGITSPSLERIYLGPGSVIDMDRLLEEYLALPRQVELWVHQNAAVVLQEHRDEEAAG
GLAPGSTRSGAGSAFIAKIRRRPGTLLFGEAVRDHPLHGVVRVVDTRTAQDMLFRTRSIQAEGC
QGYSLSVHHGAYPYCTARDVTTAQLIADCGLPYDVARIARVVGSMRTYPIRVANRPEAGEWSG
PCYPDSVECQFADLGLEQEYTTVTKLPRRIFTFSAIQAHEAIAQNGVDEVFLNFAQYPPSLGALE
DILDAIEARAEVTYVGFGPKVTDVYHTPTRAELEGLYARYRR

>1CIB_1|Chain A|ADENYLOSUCCINATE SYNTHETASE|Escherichia coli (83333)
GNNVVVLGTQWGDEGKGKIVDLLTERAKYVVRYQGGHNAGHTLVINGEKTVLHLIPSGILRE
NVTSIIGNGVVLSPAALMKEMKELEDRGIPVRERLLLSEACPLILDYHVALDNAREKARGAKAI
GTTGRGIGPAYEDKVARRGLRVGDLFDKETFAEKLKEVMEYHNFQLVNYYKAEAVDYQKVLD
DTMAVADILTSMVVDVSDLLDQARQRGDFVMFEGAQGTLLDIDHGTYPYVTSSNTTAGGVAT
GSGLGPRYVDYVLGILKAYSTRVGAGPFPTELFDETGEFLCKQGNEFGATTGRRRRTGWLDTV
AVRRAVQLNSLSGFCLTKLDVLDGLKEVKLCVAYRMPDGREVTTTPLAADDWKGVEPIYETM
PGWSESTFGVKDRSGLPQAALNYIKRIEELTGVPIDIISTGPDRTETMILRDPFDA

Supplemental Tables

Table S1 Details of the virus involved in the article

**Taxon**	**Genome size (kb)**	**Accession number**	**Phage family**	**Reference (doi)**
***Alteromonas* phage**				
JH01	46.5	MH445500	*Siphoviridae*	10.1007/s00284-019-01751-3
P24	33.5	MK241539	*Siphoviridae*	10.1007/s00284-020-02077-1
phiAFP1	5.8	MT975991	*unclassified*	Unpublished
PB15	37.3	KX982260	*Siphoviridae*	10.1007/s00284-017-1251-9
vB_AcoS-R7M	56.1	NC048878	*Siphoviridae*	0.1128/mSphere.00454-21
vB_AmaP_AD45-P1	103.9	NC021532	*Podoviridae*	10.4161/bact.24766
vB_AmaP_AD45-P2	104.4	KF005320	*Podoviridae*	10.4161/bact.24766
vB_AmaP_AD45-P3	101.7	KF005318	*Podoviridae*	10.4161/bact.24766
vB_AmaP_AD45-P4	100	KF005319	*Podoviridae*	10.4161/bact.24766
vB_AmeM_PT11-V22	92.7	NC048847	*Myoviridae*	10.1128/mSystems.00217-20
vB_AmeP_R8W	48.8	MW043865	*Autographiviridae*	10.3390/v13060987
vB_AspP-H4/4	47.6	NC047834.	*Autographiviridae*	10.1128/genomeA.00914-17
XX1924	40.5	MN592896	*Siphoviridae*	Unpublished
***Pelagibacter* phage**				
HMO-2011	55.2	NC021864	*Podoviridae*	10.1073/pnas.1219930110
HTVC010P	39.9	NC020481	*Podoviridae*	10.1038/nature11921
HTVC011P	34.8	NC020482	*Podoviridae*	10.1038/nature11921
***Prochlococcus* phage**				
P-SSP7	45.1	NC006882	*Podoviridae*	10.1371/journal.pbio.0030144
P-SSM7	182.2	NC015290	*Myoviridae*	10.1111/j.1462-
***Synechococcus* phage**				
S-SM2	190.8	NC015279	*Myoviridae*	10.1111/j.1462-
S-CBS2	72.3	NC015463	*Siphoviridae*	
***Roseobacter* phage**				
ISO1	39.9	NC002519	*Podoviridae*	10.4319/lo.2000.45.2.0408
***Vibrio* phage**				
VP2	39.8	NC005879	*Podoviridae*	
VP5	39.7	NC005891	*Podoviridae*	
phiVC8	39.4	NC027118	*Podoviridae*	10.1186/s12985-016-0490-x
QH	39.7	NC027397	*Podoviridae*	10.1128/genomeA.01019-17
J2	39.5	NC027393	*Podoviridae*	10.1128/genomeA.01019-17

Table S2 *Mareflavirus* in the IMG/VR database (shared genes > 50% with ZP6)

UViG	Length	Taxonomic classification	Sequence origin (doi)	MIUViG quality
**DTR_890774**	37675	Podovirida	CheckV (10.1101/2020.05.06.081778)	Reference
**Station178_SUR_ALL_assembly_NODE_1742_length_37730_cov_53.725786**	37730	Podovirida	GOV2 (10.1016/j.cell.2019.03.040)	High-quality
**Station180_SUR_ALL_assembly_NODE_2452_length_37730_cov_48.125680**	37730	Podovirida	GOV2 (10.1016/j.cell.2019.03.040)	High-quality
**Station180_ZZZ_ALL_assembly_NODE_1388_length_37730_cov_17.950816**	37730	Podovirida	GOV2 (10.1016/j.cell.2019.03.040)	High-quality
**Station188_DCM_ALL_assembly_NODE_1177_length_37730_cov_13.274851**	37730	Podovirida	GOV2 (10.1016/j.cell.2019.03.040)	High-quality

Table. S3 Genomic annotation of *Mareflavirus*

ORF	Start	Stop	Strand	Function	Accession	E-value
**DTR_890774**						
2	268	2331	-	structural protein	AZS06564.1	3e-156
6	5,509	5,689	-	major tail subunit	AZS06560.1	9e-34
8	6,506	6,913	-	major capsid protein	QGH73631.1	3e-133
11	8,180	9,823	-	portal protein	AWD90773.1	0.0
12	9,813	11,171	-	terminase large subunit	AZS06554.1	0.0
14	11,626	12,108	-	terminase small subunit	QMS42075.1	2e-50
25	17,119	17,658	+	3'-5' exoribonuclease	NLD09310.1	2e-53
27	18,458	19,969	+	DNA helicase	ASV42813.1	2e-144
30	21,382	21,657	+	single-stranded DNA-binding protein	AZS06529.1	6e-23
31	21,709	23,589	+	DNA polymerase	AZS06528.1	0.0
32	23,586	25,892	+	integrase	AZS06527.1	0.0
33	25,964	27,001	+	adenylosuccinate synthetase	AZS06526.1	5e-128
35	27,264	27,701	+	metal dependent phosphohydrolase	YP_009152776.1	2e-19
38	28,416	28,898	+	lysozyme	MBO2511972.1	3e-21
**Station178_SUR_ALL_assembly_NODE_1742_length_37730_cov_53.725786**						
2	376	2439	-	structural protein	AZS06564.1	3e-156
6	5,207	5,797	-	major tail subunit	AZS06560.1	9e-34
8	7,149	8,072	-	major capsid protein	QGH73631.1	4e-131
11	8,288	9,931	-	portal protein	AWD90773.1	0.0
12	9,921	11,279	-	terminase large subunit	AZS06554.1	0.0
14	11,734	12,216	-	terminase small subunit	QMS42075.1	9e-45
25	17,227	17,766	+	3'-5' exoribonuclease	NLD09310.1	2e-53
27	18,566	20,077	+	DNA helicase	ASV42813.1	2e-144
30	21,490	21,765	+	single-stranded DNA-binding protein	AZS06529.1	6e-23
31	21,817	23,697	+	DNA polymerase	AZS06528.1	0.0
32	23,694	26,000	+	integrase	AZS06527.1	0.0
33	26,072	27,109	+	adenylosuccinate synthetase	AZS06526.1	5e-128
35	27,372	27,809	+	metal dependent phosphohydrolase	YP_009152776.1	2e-19
38	28,524	29,006	+	lysozyme	MBO2511972.1	3e-21
**Station180_SUR_ALL_assembly_NODE_2452_length_37730_cov_48.125680**						
1	492	974	+	terminase small subunit	QMS42075.1	9e-45
3	1,429	2,787	+	terminase large subunit	AZS06554.1	0.0
4	2,777	4,420	+	portal protein	AWD90773.1	0.0

7	5,687	6,544	+	major capsid protein	QGH73631.1	4e-131
9	6,911	7,501	+	major tail subunit	AZS06560.1	9e-34
13	10,269	12,332	+	structural protein	AZS06564.1	3e-156
18	21,377	21,859	-	lysozyme	MBO2511972.1	3e-21
21	22,574	23,011	-	metal dependent phosphohydrolase	YP_009152776.1	2e-19
23	23,274	24,311	-	adenylosuccinate synthetase	AZS06526.1	5e-128
24	24,383	26,689	-	integrase	AZS06527.1	0.0
25	26,686	28,566	-	DNA polymerase	AZS06528.1	0.0
26	28,618	28,893	-	single-stranded DNA-binding protein	AZS06529.1	6e-23
29	30,306	31,817	-	DNA helicase	ASV42813.1	2e-144
31	32,617	33,156	-	3'-5' exoribonuclease	NLD09310.1	2e-53
**Station180_ZZZ_ALL_assembly_NODE_1388_length_37730_cov_17.950816**						
2	268	2331	-	structural protein	AZS06564.1	3e-156
6	5099	5689	-	major tail subunit	AZS06560.1	9e-34
8	6056	6913	-	major capsid protein	QGH73631.1	4e-131
11	8180	9823	-	portal protein	AWD90773.1	0.0
12	9813	11171	-	terminase large subunit	AZS06554.1	0.0
14	11626	12108	-	terminase small subunit	QMS42075.1	9e-45
25	17119	17658	+	3'-5' exoribonuclease	NLD09310.1	2e-53
27	18458	19969	+	DNA helicase	ASV42813.1	2e-144
30	21382	21657	+	single-stranded DNA-binding protein	AZS06529.1	6e-23
31	21709	23589	+	DNA polymerase	AZS06528.1	0.0
32	23586	25892	+	integrase	AZS06527.1	0.0
33	25964	27001	+	adenylosuccinate synthetase	AZS06526.1	5e-128
35	27264	27701	+	metal dependent phosphohydrolase	YP_009152776.1	2e-19
38	28416	28898	+	lysozyme	MBO2511972.1	3e-21
**Station188_DCM_ALL_assembly_NODE_1177_length_37730_cov_13.274851**						
1	492	974	+	terminase small subunit	QMS42075.1	9e-45
3	1429	2787	+	terminase large subunit	AZS06554.1	0.0
4	2777	4420	+	portal protein	AWD90773.1	0.0
7	5687	6544	+	major capsid protein	QGH73631.1	4e-131
9	6911	7501	+	major tail subunit	AZS06560.1	9e-34
13	10269	12332	+	structural protein	AZS06564.1	3e-156
18	21377	21859	-	lysozyme	MBO2511972.1	3e-21
21	22574	23011	-	metal dependent phosphohydrolase	YP_009152776.1	2e-19
23	23274	24311	-	adenylosuccinate synthetase	AZS06526.1	5e-128

24	24383	26689	-	integrase	AZS06527.1	0.0
25	26686	28566	-	DNA polymerase	AZS06528.1	0.0
26	28618	28893	-	single-stranded DNA-binding protein	AZS06529.1	6e-23
29	30306	31817	-	DNA helicase	ASV42813.1	2e-144
31	32617	33156	-	3'-5' exoribonuclease	NLD09310.1	2e-53

Table S4

Identified PurZ (diaminopurine synthetase) and typical adenylosuccinate synthetase (PurA)

Proteins	Accession	Organism	Domain
SbPurZ	TXG97710.1	*Sinobacteraceae bacterium*	Bacterium
SpPurZ	AUZ95522.1	*Salmonella* phage PMBT28	Phage
ApPurZ	AUG85481.1	*Acinetobacter* phage SH-Ab 15497	Phage
VpPurZ	YP009140156.1	*Vibrio* phage phiVC8	Phage
ZpPurZ	AZS06526.1	*Alteromonas* phage ZP6	Phage
CpPurZ	QQG31319.1	cyanophage S-2L	Phage
EcPurA	1CIB_1 (PDB)	*Escherichia coli*	Bacterium

The data are from the article published in Science by Yan Zhou et al. in 30 Apr, 2021 (1).

References

1. Zhou Y, Xu X, Wei Y, Cheng Y, Guo Y, Khudyakov I, Liu F, He P, Song Z, Li Z, Gao Y, Ang EL, Zhao H, Zhang Y, Zhao S. 2021. A widespread pathway for substitution of adenine by diaminopurine in phage genomes. Science 372:512-516.

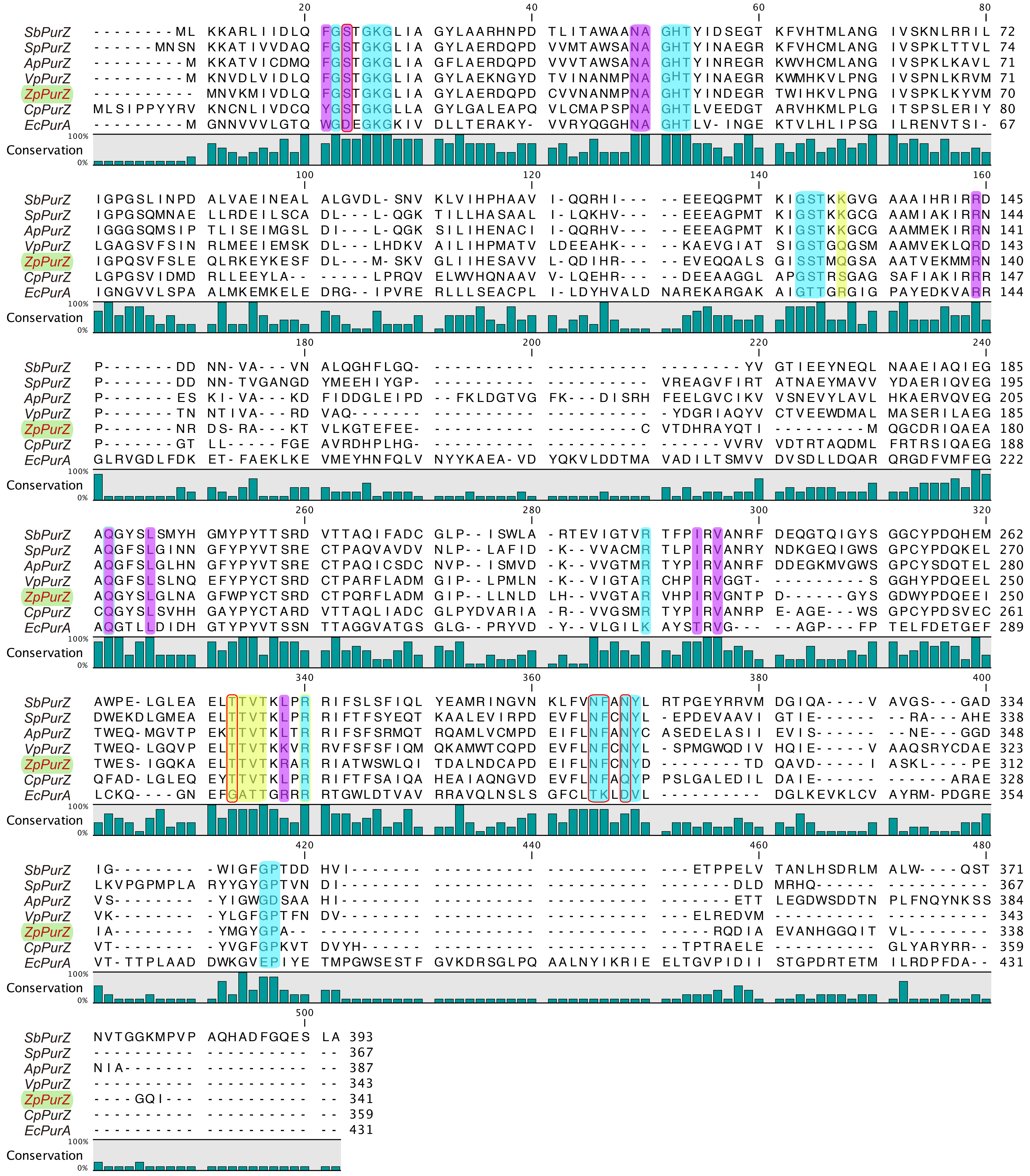
20 40 60 80
SbPurZ --------ML KKARLIIDLQ FGSTGKGLIA GYLAARHNPD TLITAWAANA GHTYIDSEGT KFVHTMLANG IVSKNLRRIL 72
SpPurZ ------MNSN KKATIVVDAQ FGSTGKGLIA GYLAERDQPD VVMTAWSANA GHTYINAEGR KFVHCMLANG IVSPKLTTVL 74
ApPurZ ---------M KKATVICDMQ FGSTGKGLIA GFLAERDQPD VVVTAWSANA GHTYINREGR KWVHCMLANG IVSPKLKAVL 71
VpPurZ ---------M KNVDLVIDLQ FGSTGKGLIA GYLAEKNGYD TVINANMPNA GHTYINAEGR KWMHKVLPNG IVSPNLKRVM 71
ZpPurZ ---------- MNVKMIVDLQ FGSTGKGLIA GYLAERDQPD CVVNANMPNA GHTYINDEGR TWIHKVLPNG IVSPKLKYVM 70
CpPurZ MLSIPPYYRV KNCNLIVDCQ YGSTGKGLLA GYLGALEAPQ VLCMAPSPNA GHTLVEEDGT ARVHKMLPLG ITSPSLERIY 80
EcPurA ---------M GNNVVVLGTQ WGDEGKGKIV DLLTERAKY- VVRYQGGHNA GHTLV-INGE KTVLHLIPSG ILRENVTSI- 67
100%
Conservation
0%
100 120 140 160
SbPurZ IGPGSLINPD ALVAEINEAL ALGVDL-SNV KLVIHPHAAV I-QQRHI--- --EEEQGPMT KIGSTKKGVG AAAIHRIRRD 145
SpPurZ IGPGSQMNAE LLRDEILSCA DL---L-QGK TILLHASAAL I-LQKHV--- --EEEAGPMT KIGSTKKGCG AAMIAKIRRN 144
ApPurZ IGGGSQMSIP TLISEIMGSL DI---L-QGK SILIHENACI I-QQRHV--- --EEEAGPMT KIGSTKKGCG AAMMEKIRRN 141
VpPurZ LGAGSVFSIN RLMEEIEMSK DL---LHDKV AILIHPMATV LDEEAHK--- --KAEVGIAT SIGSTGQGSM AAMVEKLQRD 143
ZpPurZ IGPQSVFSLE QLRKEYKESF DL---M-SKV GLIIHESAVV L-QDIHR--- --EVEQQALS GISSTMQGSA AATVEKMMRN 140
CpPurZ LGPGSVIDMD RLLEEYLA-- -----LPRQV ELWVHQNAAV V-LQEHR--- --DEEAAGGL APGSTRSGAG SAFIAKIRRR 147
EcPurA IGNGVVLSPA ALMKEMKELE DRG--IPVRE RLLLSEACPL I-LDYHVALD NAREKARGAK AIGTTGRGIG PAYEDKVARR 144
100%
Conservation
0%
180 200 220 240
SbPurZ P-------DD NN-VA---VN ALQGHFLGQ- ---------- ---------- --------YV GTIEEYNEQL NAAEIAQIEG 185
SpPurZ P-------DD NN-TVGANGD YMEEHIYGP- ---------- ---------- VREAGVFIRT ATNAEYMAVV YDAERIQVEG 195
ApPurZ P-------ES KI-VA---KD FIDDGLEIPD --FKLDGTVG FK---DISRH FEELGVCIKV VSNEVYLAVL HKAERVQVEG 205
VpPurZ P-------TN NTIVA---RD VAQ------- ---------- ---------- -YDGRIAQYV CTVEEWDMAL MASERILAEG 185
ZpPurZ P-------NR DS-RA---KT VLKGTEFEE- ---------- ---------C VTDHRAYQTI ---------M QGCDRIQAEA 180
CpPurZ P-------GT LL-----FGE AVRDHPLHG- ---------- ---------- ------VVRV VDTRTAQDML FRTRSIQAEG 188
EcPurA GLRVGDLFDK ET-FAEKLKE VMEYHNFQLV NYYKAEA-VD YQKVLDDTMA VADILTSMVV DVSDLLDQAR QRGDFVMFEG 222
100%
Conservation
0%
260 280 300 320
SbPurZ AQGYSLSMYH GMYPYTTSRD VTTAQIFADC GLP--ISWLA -RTEVIGTVR TFPIRVANRF DEQGTQIGYS GGCYPDQHEM 262
SpPurZ AQGFSLGINN GFYPYVTSRE CTPAQVAVDV NLP--LAFID -K--VVACMR TLPIRVANRY NDKGEQIGWS GPCYPDQKEL 270
ApPurZ AQGFSLGLHN GFYPYVTSRE CTPAQICSDC NVP--ISMVD -K--VVGTMR TYPIRVANRF DDEGKMVGWS GPCYSDQTEL 280
VpPurZ AQGFSLSLNQ EFYPYCTSRD CTPARFLADM GIP--LPMLN -K--VIGTAR CHPIRVGGT- ---------S GGHYPDQEEL 250
ZpPurZ AQGYSLGLNA GFWPYCTSRD CTPQRFLADM GIP--LLNLD LH--VVGTAR VHPIRVGNTP D------GYS GDWYPDQEEI 250
CpPurZ CQGYSLSVHH GAYPYCTARD VTTAQLIADC GLPYDVARIA -R--VVGSMR TYPIRVANRP E-AGE---WS GPCYPDSVEC 261
EcPurA AQGTLLDIDH GTYPYVTSSN TTAGGVATGS GLG--PRYVD -Y--VLGILK AYSTRVG--- --AGP---FP TELFDETGEF 289
100%
Conservation
0%
340 360 380 400
SbPurZ AWPE-LGLEA ELTTVTKLPR RIFSLSFIQL YEAMRINGVN KLFVNFANYL RTPGEYRRVM DGIQA----V AVGS---GAD 334
SpPurZ DWEKDLGMEA EKTTVTKLPR RIFTFSYEQT KAALEVIRPD EVFLNFCNYL -EPDEVAAVI GTIE------ --RA---AHE 338
ApPurZ TWEQ-MGVTP EKTTVTKLPR RIFSFSRMQT RQAMLVCMPD EIFLNFCNYC ASEDELASII EVIS------ --NE---GGD 348
VpPurZ TWEQ-LGQVP ELTTVTKKVR RVFSFSFIQM QKAMWTCQPD EVFLNFCNYL -SPMGWQDIV HQIE-----V AAQSRYCDAE 323
ZpPurZ TWES-IGQKA ELTTVTKRAR RIATWSWLQI TDALNDCAPD EIFLNFCNYD --------TD QAVD-----I ASKL----PE 312
CpPurZ QFAD-LGLEQ EYTTVTKLPR RIFTFSAIQA HEAIAQNGVD EVFLNFAQYP PSLGALEDIL DAIE------ ------ARAE 328
EcPurA LCKQ----GN EFGATTGRRR RTGWLDTVAV RRAVQLNSLS GFCLTKLDVL ---------- DGLKEVKLCV AYRM-PDGRE 354
100%
Conservation
0%
420 440 460 480
SbPurZ IG-------- -WIGFGPTDD HVI------- ---------- ---------- ---ETPPELV TANLHSDRLM ALW----QST 371
SpPurZ LKVPGPMPLA RYYGYGPTVN DI-------- ---------- ---------- -------DLD MRHQ------ ---------- 367
ApPurZ VS-------- -YIGWGDSAA HI-------- ---------- ---------- -------ETT LEGDWSDDTN PLFNQYNKSS 384
VpPurZ VK-------- -YLGFGPTFN DV-------- ---------- ---------- ---ELREDVM ---------- ---------- 343
ZpPurZ IA-------- -YMGYGPA-- ---------- ---------- ---------- -----RQDIA EVANHGGQIT VL-------- 338
CpPurZ VT-------- -YVGFGPKVT DVYH------ ---------- ---------- --TPTRAELE ---------- GLYARYRR-- 359
EcPurA VT-TTPLAAD DWKGVEPIYE TMPGWSESTF GVKDRSGLPQ AALNYIKRIE ELTGVPIDII STGPDRTETM ILRDPFDA-- 431
100%
Conservation
0%
500
SbPurZ NVTGGKMPVP AQHADFGQES LA 393
SpPurZ ---------- ---------- -- 367
ApPurZ NIA------- ---------- -- 387
VpPurZ ---------- ---------- -- 343
ZpPurZ ----GQI--- ---------- -- 341
CpPurZ ---------- ---------- -- 359
EcPurA ---------- ---------- -- 431
100%
Conservation
0%

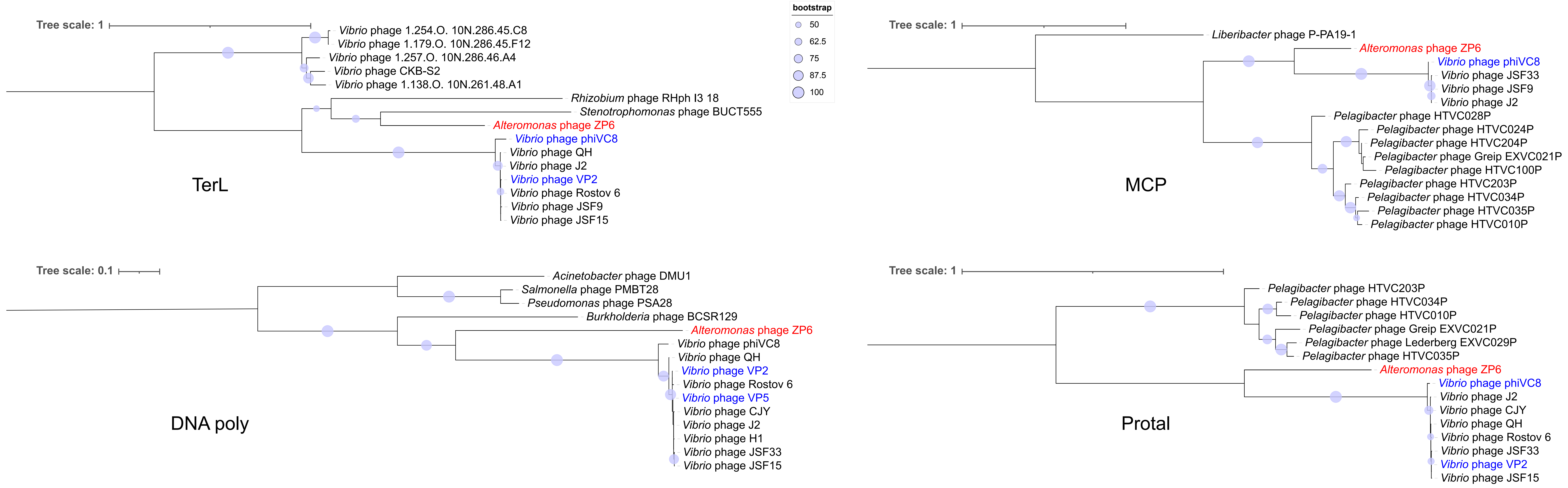
Tree scale: 1
Vibrio phage 1.254.O. 10N.286.45.C8
Vibrio phage 1.179.O. 10N.286.45.F12
Vibrio phage 1.257.O. 10N.286.46.A4
Vibrio phage CKB-S2
Vibrio phage 1.138.O. 10N.261.48.A1
Rhizobium phage RHph I3 18
Stenotrophomonas phage BUCT555
Alteromonas phage ZP6
Vibrio phage phiVC8
Vibrio phage QH
Vibrio phage J2
Vibrio phage VP2
Vibrio phage Rostov 6
Vibrio phage JSF9
Vibrio phage JSF15
TerL
bootstrap
50
62.5
75
87.5
100
Tree scale: 1
Liberibacter phage P-PA19-1
Alteromonas phage ZP6
Vibrio phage phiVC8
Vibrio phage JSF33
Vibrio phage JSF9
Vibrio phage J2
Pelagibacter phage HTVC028P
Pelagibacter phage HTVC024P
Pelagibacter phage HTVC204P
Pelagibacter phage Greip EXVC021P
Pelagibacter phage HTVC100P
Pelagibacter phage HTVC203P
Pelagibacter phage HTVC034P
Pelagibacter phage HTVC035P
Pelagibacter phage HTVC010P
MCP
Tree scale: 0.1
Acinetobacter phage DMU1
Salmonella phage PMBT28
Pseudomonas phage PSA28
Burkholderia phage BCSR129
Alteromonas phage ZP6
Vibrio phage phiVC8
Vibrio phage QH
Vibrio phage VP2
Vibrio phage Rostov 6
Vibrio phage VP5
Vibrio phage CJY
Vibrio phage J2
Vibrio phage H1
Vibrio phage JSF33
Vibrio phage JSF15
DNA poly
Tree scale: 1
Pelagibacter phage HTVC203P
Pelagibacter phage HTVC034P
Pelagibacter phage HTVC010P
Pelagibacter phage Greip EXVC021P
Pelagibacter phage Lederberg EXVC029P
Pelagibacter phage HTVC035P
Alteromonas phage ZP6
Vibrio phage phiVC8
Vibrio phage J2
Vibrio phage CJY
Vibrio phage QH
Vibrio phage Rostov 6
Vibrio phage JSF33
Vibrio phage VP2
Vibrio phage JSF15
Protal

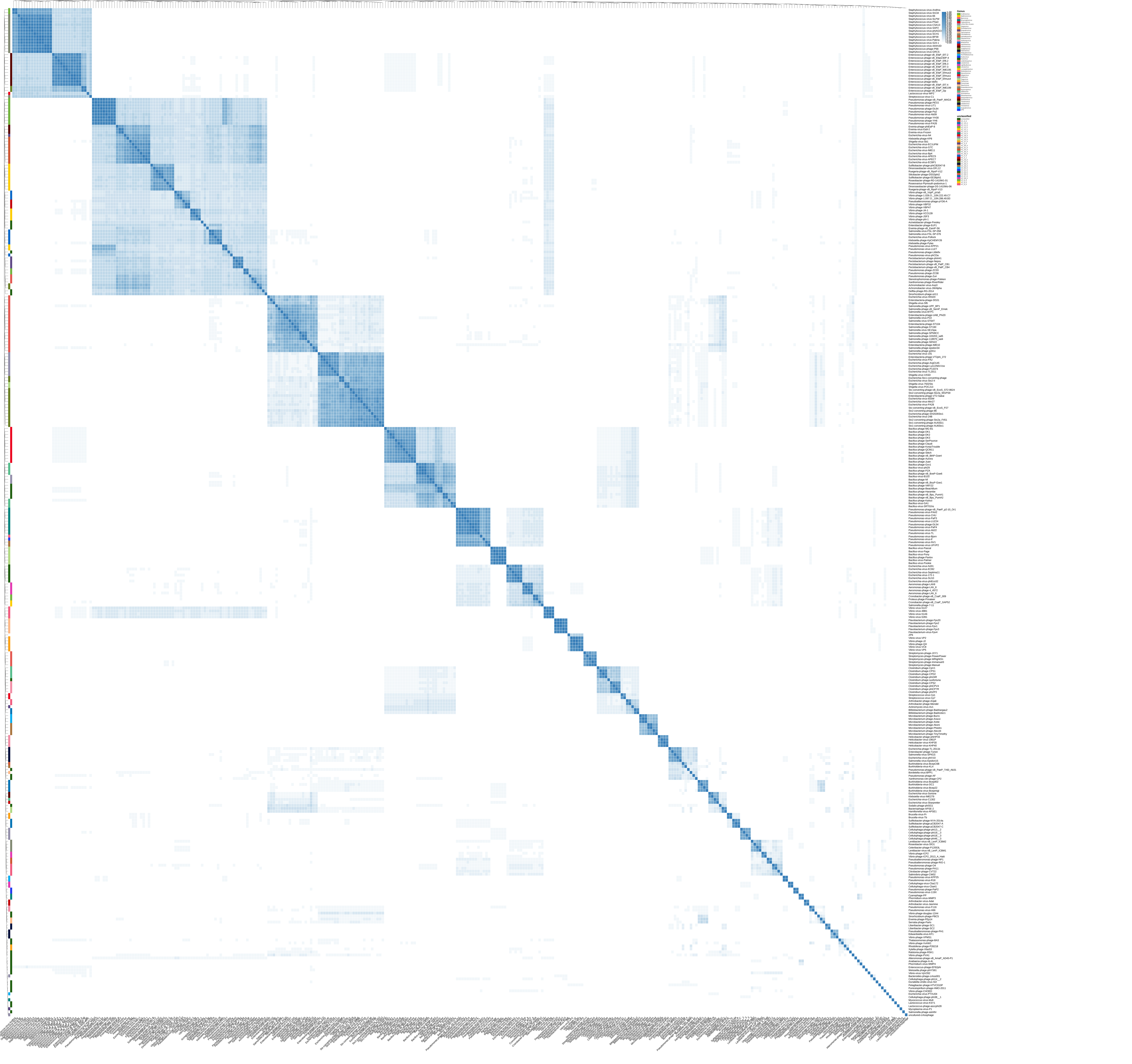

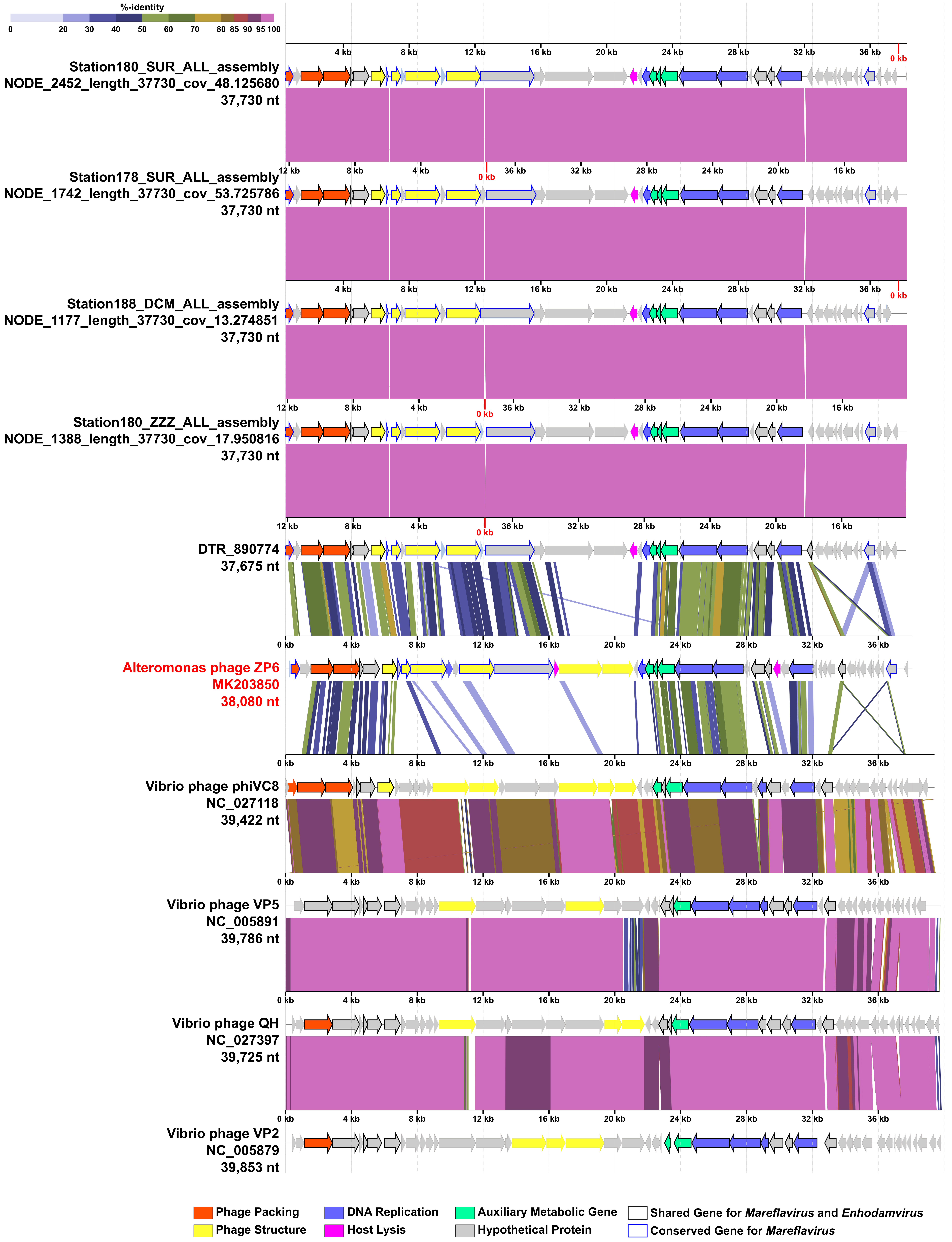

%-identity
0
20
30
40
50
60
70
80
85
90
95
100
Station180_SUR_ALL_assembly
NODE_2452_length_37730_cov_48.125680
37,730 nt
Station178_SUR_ALL_assembly
NODE_1742_length_37730_cov_53.725786
37,730 nt
Station188_DCM_ALL_assembly
NODE_1177_length_37730_cov_13.274851
37,730 nt
Station180_ZZZ_ALL_assembly
NODE_1388_length_37730_cov_17.950816
37,730 nt
DTR_890774
37,675 nt
Alteromonas phage ZP6
MK203850
38,080 nt
Vibrio phage phiVC8
NC_027118
39,422 nt
Vibrio phage VP5
NC_005891
39,786 nt
Vibrio phage QH
NC_027397
39,725 nt
Vibrio phage VP2
NC_005879
39,853 nt
0 kb
4 kb
8 kb
12 kb
16 kb
20 kb
24 kb
28 kb
32 kb
36 kb
Phage Packing
Phage Structure
DNA Replication
Host Lysis
Auxiliary Metabolic Gene
Hypothetical Protein
Shared Gene for *Mareflavirus* and *Enhodamvirus*
Conserved Gene for *Mareflavirus*
